# Case Studies on The Use of LiveLink for MATLAB for Evaluation and Optimization of The Heat Sources in Experimental Borehole

**DOI:** 10.3390/s20051297

**Published:** 2020-02-27

**Authors:** Stepan Ozana, Radovan Hajovsky, Martin Pies, Radek Martinek

**Affiliations:** Faculty of Electrical Engineering and Computer Science, Department of Cybernetics and Biomedical Engineering, VŠB–Technical University of Ostrava, 708 00 Ostrava-Poruba, Czech Republic; radovan.hajovsky@vsb.cz (R.H.); martin.pies@vsb.cz (M.P.); radek.martinek@vsb.cz (R.M.)

**Keywords:** borehole heat exchanger (BHE), COMSOL multiphysics, liveLink for MATLAB, MATLAB, optimization, partial differential equation

## Abstract

In the Czech part of the Upper Silesian Coal Basin (Moravian-Silesian region, Czech Republic), there are many deposits of endogenous combustion (e.g., localized burning soil bodies, landfills containing industrial waste, or slag rocks caused by mining processes). The Hedwig mining dump represents such an example of these sites where, besides the temperature and the concentrations of toxic gases, electric and non-electric quantities are also monitored within the frame of experimentally proposed and patented technology for heat collection (the so-called “Pershing” system). Based on these quantities, this paper deals with the determination and evaluation of negative heat sources and the optimization of the positive heat source dependent on measured temperatures within evaluation points or on a thermal profile. The optimization problem is defined based on a balance of the heat sources in the steady state while searching for a local minimum of the objective function for the heat source. From an implementation point of view, it is the interconnection of the numerical model of the heat collector in COMSOL with a user optimization algorithm in MATLAB using the LiveLink for MATLAB. The results are elaborated in five case studies based on the susceptibility testing of the numerical model by input data from the evaluation points. The tests were focused on the model behavior in terms of preprocessing for measurement data from each chamber of the heat collector and for the estimated value of temperature differences at 90% and 110% of the nominal value. It turned out that the numerical model is more sensitive to the estimates in comparison with the measured data of the chambers, and this finding does not depend on the type optimization algorithm. The validation of the model by the use of the mean-square error led to the finding of optimal value, also valid with respect to the other evaluation.

## 1. Introduction

In the Czech Republic, the initiation of combustion at dumps is usually related either to setting a fire at a dump itself or transferring the hot ash from incinerators at a waste dump. Furthermore, according to [1], the initiation of such fires was subject to the existence of mature self-seeded trees with a sufficiently developed root system, while the fire itself was initiated through this flora system. In the Czech part of the Upper Silesian Basin, these fires pose a serious ecological problem (see [2,3]); the analysis of these documented cases shows the main reasons of the initiation: Setting up an open fire at the surface, spontaneous combustion of the waste rock, gob fire, and improper opening of the dump during its excavation.

Abroad, there can be a wider variety of possible causes of ignition by summer forest fires in 2005 (the Portuguese coal region São Pedro da Cova in the Porto district, or Pejão in the Aveiro district, both in the Rio Duoro river basin; see [4]), a lightning ignition or ignition due to carelessness when handling an open fire, or initiation due to solar rays. The latter possibility usually occurs in countries with a high average temperature where coal seams are present on the surface of mountains or desert steppes [for example, Shanxi province in China (see [5,6,7,8]), India, the Republic of South Africa, Kentucky in the USA (see [9]), Russian Federation, etc.] [10] shows the composition of extracted waste rock. A similar issue is analyzed with [11], where the principle of air-conditioning of these mines is described using a mathematical model of a deep mine. It shows the mathematical model and calculation of the temperature balance. Finally, the results of the experiment are added, where the excess heat from the mine is transferred to the water reservoirs. The opposite case is considered in [12], which describes the heating of traffic tunnels excavated in permafrost using heating cables. In terms of the development prognosis, the ongoing combustion represents a thermal process involving 5 stages; according to [13], the individual stages may be terminated at any time, but the stage concerning progressive burnout (related to the cooling below +75 °C) is inevitable and always present. The consequences of the burning dump are not only bound to a specific period of active burning, being related to the dump’s entire existence in the countryside. [13] also mentions the problem of the localization of centers of the fires at mining dumps and abandoned underground mining facilities (for example, an analysis of gases evaporated from coal substance, an indication of gob fire through the ratio of the volumes of hydrocarbons, etc.). In the case of attenuation of a fire, it is possible to use technology based on prevention, status check, and extinguishing itself, for example with the use of a combination of Portland cement, fly ash, aggregate, special foam, or injection into the hot zones within coal slurry using liquid nitrogen at −180 °C.

Research on burning mining dumps not only aims at localizing centers of fires but also at identifying crucial environmental contaminants and other environmental risks. Finally, the survey also focuses on the use of burnt-down tailings such as in the construction industry (see [14]).

Of course, there arises a question of the possibility of simulation of thermal processes at coal seams and mining dumps. [15] introduces, in general features, the methodology and results of simulated coal fires carried out under simplified conditions. It showed a demonstration of the differences between measured data related to these fires at terrestrial and aerial observation spots (aerial photography). Two-dimensional mathematical that modeled simulated thermal field distribution considering subsurface coal fire was demonstrated in [16].

Naturally, there is a possibility to use mathematical modeling to forecast the combustion of coal seams. [17], for example, contains the burnout of three such seams in the Upper Silesian coal basin (see also [2,3]), and which was done through nonlinear initial and boundary conditions. The calculation used results from the experiments with consideration of the thermal and oxidation alteration of rocks and coal substance. Furthermore, the model referred to in [17] also incorporated information obtained by studying the real rock and coal from solid layers and their vicinity. The particular model based on the Finite Element Method (FEM, see also [18] and the numerical model in [8]) was created in FEMLAB (predecessor of the COMSOL Multiphysics; see also [19]). The calculated range of temperatures varied between +350 °C and +800 °C, and the estimated time to burnout was approximately 2000 years.

## 2. Materials and Methods

The beginnings of the initiation of fires at dumps, established in the late 19th century and the first half of the 20th century, according to [13], are not reliably known nor adequately documented in the literature. The issue always involves determining the end of dump burning itself (mainly the effects of active fire and the presence of invasive trees with a root system). From a practical point of view, the duration of the burning of such materials can only be guessed at, e.g., in the case of medium-sized mining at the Ema dump (324.3 m; Silesian Ostrava), located near the Hedwig dump, the estimated burning time is approximately 50–60 years.

In the Czech part of the Upper Silesian Coal Basin (i.e., the Moravian-Silesian Region), there have been various historically applied remediation technologies (e.g., excavation of the source of fire and subsequent cooling). [10] gives a summary of redeveloped localities within Ostrava (for example, the Hrabůvka dump in Ostrava-Hrabůvka, the Zárubek mining dump in Ostrava-Kunčičky, the railway embankment in the site of the Moravian chemical plant in Ostrava–Mariánské Hory, the isolated burning body of the railway embankment in the Heřmanice dump in Ostrava-Heřmanice) and close surroundings (for example, the Vrbice dump in Bohumín-Vrbice). [14] also introduces the redeveloped locality outside the Moravian-Silesian region (for example, the Krimich dump in Tlučná in the Pilsen region).

The first mathematical model of the flow of air in the thermally active zones Heřmanice and Hedwig (within the Ostrava-Karvina coal mining region) was created in 2004 to confront the results of the model with exploration boreholes (see [17]). The paper [1] shows graphical isotherms over three months (August to November) within active zones of burning dump Hedwig, in depths of 3 m and 6 m, for the period from August 2011 to November 2011. The highest temperature was recorded in December 2011, when the temperature at a depth of 6 m reached +500 °C.

### 2.1. The Hedwig Mining Dump

Another option when monitoring thermal processes not only in the dumps but also in mine disposal sites and landfills are temperature (or thermal) monitoring, whose main purpose is the study of thermal fields and especially the forecast of its development within a certain timeframe. Let us add that according to [20] thermal monitoring represents an integral part of the exploration work on thermally active sites; each group of stages, according to [20], can be divided into stages regarding the exploratory survey and detailed surveys of the area (see also [8]). The prototype of the thermic monitoring system at the Hedwig mining dump was already installed in 2012 (see also Table 1).

**Figure 1:**
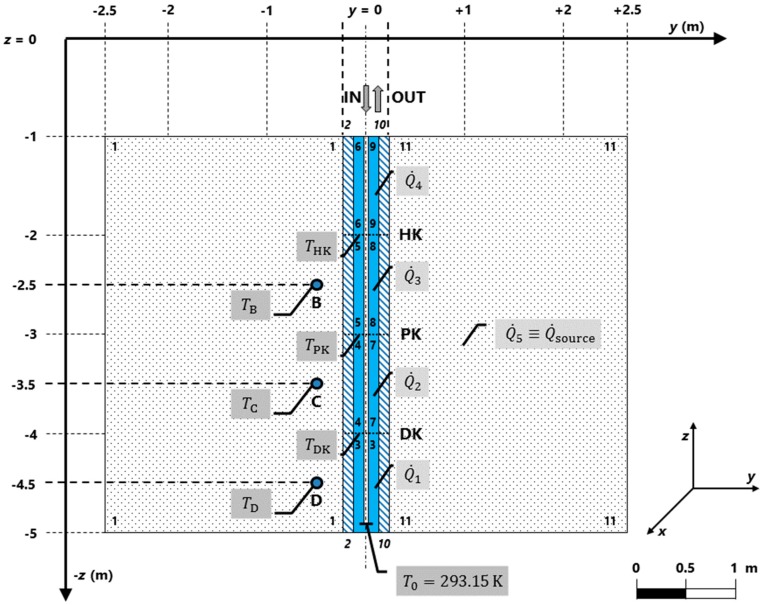
2D conceptual model: location of the domains and their notification, heat sources, initial value of thermodynamic temperature, and chosen coordinate system (*yz-plane*).

This telemetry system represents a unique technology to a certain extent; it is capable of measuring and logging temperatures and concentrations of toxic gases (methane CH_4_ and carbon monoxide CO), as well as other electric and non-electric signals within the frame of the experimentally developed heat collector system (the “Pershing” system; see also Figure 1 and [21]).

The original idea used in the created mathematical model of the heat collector for the site was based on a balance of heat flows (see also [22] and Section 2.2.1), while in the case of stationary tasks Equation (2) can be considered. On the 26th of November 2013, this idea, entitled “Apparatus for measuring temperature fields in rock massif” was registered at the Czech Industrial Property Office in Prague (reg. no. 2013–936). The particular patent was rewarded on the 22nd of July 2015 and published on the 2nd of September 2015.

According to [23], the heat exchanger represents the basic element of the device for the measurement of thermal fields in a rock massif. This heat exchanger is divided into four sections (see also Figure 1) with the use of bulkheads with holes, fitted with temperature sensors connected to the communication bus. The top of the heat exchanger contains an inlet (media input point) and a reverse (output media point). At given distances from the heat exchanger, many measuring probes are installed (see also Figure 1).

**Figure 2:**
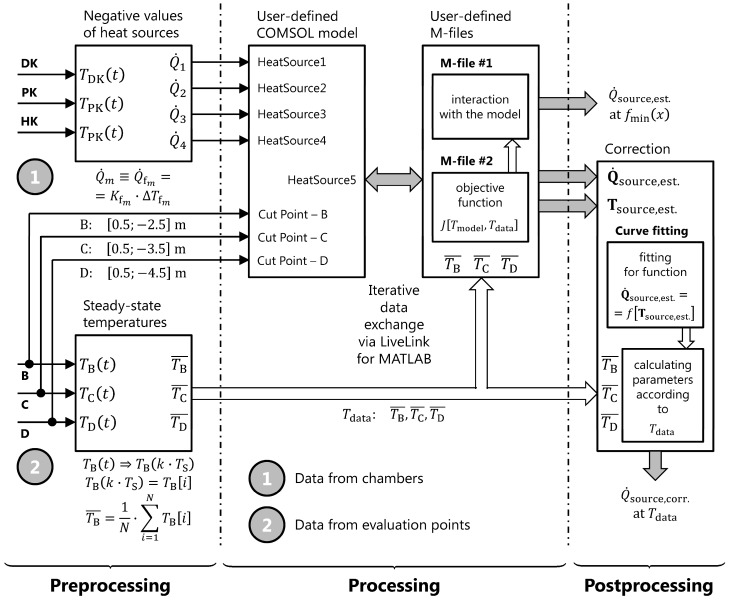
Concept of the solution of the stationary job—block scheme and data flow diagram of the preprocessing (treating input data from chambers and evaluation points), processing (iteration data exchange between the COMSOL model and Matlab custom code via the use of the LiveLink for MATLAB), and postprocessing (treating output data depending on the optimization type) parts.

Considering the (simplified) mathematical model, this paper primarily discusses a two-dimensional model (i.e., 2D model) created in COMSOL Multiphysics (version 4.3; build 151). Regarding the benefit of this paper, the introduced 2D model of the “Pershing” system demonstrates the need to optimize the value of the heat source, based on the minimization of the cost function involving the optimization criteria [see Equations (16) and (17)], actually measured data (from the chambers and selected evaluation points; see Figure 2), data from the model, and key exchange data using LiveLink for MATLAB (i.e., between the 2D models in COMSOL and user implementation of the optimization algorithm in Matlab, see Figure 2, see [19,24]).

The objectives in addressing this issue are identical with the structure of this article and based on resolving these broader and more specific challenges, namely:

- Brief description of the existing technology of the heat collector (and its surroundings) associated with the implementation of the key findings (typically geometry and physics mathematical model in COMSOL); see Section 2.1.1 to Section 2.1.2 and Section 2.2.1, Section 2.2.2, Section 2.2.3 and Section 2.2.4.
- Design and implementation of optimization problems in Matlab code and COMSOL model (from a technical point of view, this involves linking COMSOL and MATLAB using the LiveLink for MATLAB); see Section 2.2.5.
- Evaluation of the results of the optimization problem; see Section 3.1.
- Validation of a created mathematical model to real measured data; see Section 3.2.

GIS data sources are used for so-called *geologic characteristics* (schematically shown in [25]) for the Hedwig mining dump (see [6]); see Section “Geographical Viewpoint” (for a geographic point of view), Paragraph “Geomorphological Viewpoint” (for a geomorphological point of view), and Paragraph “Geological Viewpoint” (for a geological point of view).

#### 2.1.1. Natural Conditions at the Hedwig Mining Dump

The territory where the Hedwig mining dump is located belongs, according to [26], to the outer part of the geologic province of the Western Carpathians in the geomorphological area of the Northern Out-Carpathian lowlands, which are represented by the Ostravian Basin in the Northern Moravia and Silesia with a surface area of 483.08 km^2^, medium-altitude of 244 m, and medium inclination of 1°38′ (see [27]). Regarding the character of the area, it varies from the plain to hilly areas with extensive river terraces. Coal mining in the Ostrava basin has led to the creation of many anthropogenic forms (e.g., subsidence lowlands, mounds, and pits).

The original relief on the location of the Hedwig dump is quite undulating, especially in its neighborhood which is used to store mining waste. The current relief dump is the result of landscaping and the subsequent reclamation works. The altitude on this site varies according to [28] from 277.2 m to 283.2 m.

The approximate location and altitude of the heat collector are as follows [28]:S-JTSK/Krovak East North:X = −466,020.736 mY = −1,103,047.266 mETRS89:B = 49°49′35″L = 18°21′41″Altitude:278.1 m


##### Geographical Viewpoint

From a geographic point of view, the site can be categorized as follows (according to ISO 3166-1, ISO 3166-2, CZ-NUTS 3, CZ-NUTS 4, and CZ-NUTS 5), see [28]:State:Czech Republic(CZ)Region:Moravian-Silesian Region(CZ-MO; Z080)District:Ostrava-city, Karviná(CZ0806, CZ0803)Cadastre:Radvanice a Bartovice, Petřvald u Karviné(554821, 599085)

The site of the Hedwig mining dump is located on the border between the Ostrava-Radvanice suburb and the village of Petřvald u Karviné.

##### Geomorphological Viewpoint

From the geomorphological viewpoint, this locality is categorized as follows [26]:System:Alpine-Himalayan
Province:The Western Carpathians
Sub-province:The Outer Carpathian Depressions(VIII)Sub-system:The Northern Outer Carpathian Depressions(VIIIB)Region:Ostravian basin(VIIIB-1)Sub-region:Ostravian Plateaus(VIIIB-1B)District:Orlovian Plateau(VIIIB-1B-1)

The Orlovian platform represents, according to [27], the flat surface of the hilly area, measuring 135.97 km^2^. It contains various powerful strata of Quaternary glacial gravels, sands, and clays in the overlying carboniferous substance covered with a layer of loess loam.

##### Geological Viewpoint

Regarding the geological composition from the carbon ceiling to the level of the Poruba layers, it consists of Miocene clays up to a depth of 200 m. These clays are mounted on the ceiling of weathered carbon, they are then annealed by few thick glacifluvial gray gravels (thickness of about 1 m), and are subsequently annealed by glacial yellow sands and clay-sand yellow-brown boulder clays. Loess loam of the Würmian age, forming the uppermost part of the geological cover, were silted during the reclamation work in the wider surroundings of the site.

Backfills in the form of mining waste have quite a variable thickness depending on the original vertical articulation of the surface. The average thickness of the bulk materials is 15 m, and along the axis of the main valley it can be up to 40 m (see Table 1). These backfills are made of sandstone, siltstone, and unpaved claystone blended with coal substance. In terms of granites (grain), rather smaller fractions predominate.

Over time, store tailings from the mining and rock excavation of preparatory and early development workings had been stored at the Hedwig mining dump. Concerning the technology of mining and processing of coal in the period between the 20 s and the 50 s of the 20th century, the waste rock contains a significant percentage of both coal and aleuropelites with a high content of coal matter; a more detailed structure of the individual layers is given in [10]. Despite minor excavations of small pieces of coal due to human activity, a vast amount of coal substance has stayed in the dumps. On a local scale, this percentage may be up to 50% of combustible substances. An analysis of the samples taken from stored tailings showed that at a depth of 4 m below the surface of the combustible matter, the content was 16.77% on average, while at depths between 5 m and 10 m it was 15.22% on average. Based on the results of the analysis and also according to [10], it is shown that rock waste has an almost constant content of combustibles in thermally unconverted rocks, being spread vertically without any significant changes. According to the distribution of isotherms (see appendix [10]) at the Hedwig mining dump, it can also be observed that that higher temperatures can be reached at smaller depths due to a higher proportion of combustible substances made in shallow depths (typically 3 m to 4 m).

The average composition of the waste rock at the Hedwig mining dump includes 18% sandstones (i.e., $p_{1}=0.18$, at $\lambda_{\mathrm{sandstone}}=2.00\;W\cdot m^{-1}\cdot K^{-1}$, $\rho_{\mathrm{sandstone}}=2500\;\mathrm{kg}\cdot m^{-3}$), 50% siltstones (i.e., $p_{2}=0.50$, at $\lambda_{\mathrm{siltstone}}=2.30\;W\cdot m^{-1}\cdot K^{-1}$, $\rho_{\mathrm{siltstone}}=2600\;\mathrm{kg}\cdot m^{-3}$), and 32% claystones (i.e., $p_{3}=0.32$, at $\lambda_{\mathrm{claystone}}=2.20\;W\cdot m^{-1}\cdot K^{-1}$, $\rho_{\mathrm{claystone}}=2600\;\mathrm{kg}\cdot m^{-3}$), when grouping aleuropelites: 18% sandstones an 82% aleuropelites without differentiation of stratigraphic affiliation–these simplified values are used for calculations by the use of Equation (1) according to [20] and with the values used in [11].

The particular numerical COMSOL model presumes the content of the Hedwig mining dump in the form of material parameters (for domains 1 and 11 with the target object Heat Source 5, see Table 1 and Table 2) as follows:

(1) $$\begin{array}{c} \lambda_{s}=p_{1}\cdot \lambda_{\mathrm{sandstone}}+p_{2}\cdot \lambda_{\mathrm{siltstone}}+p_{3}\cdot \lambda_{\mathrm{claystone}}\;\left(W\cdot m^{-1}\cdot K^{-1}\right)\\ \rho_{s}=p_{1}\cdot \rho_{\mathrm{sandstone}}+p_{2}\cdot \rho_{\mathrm{siltstone}}+p_{3}\cdot \rho_{\mathrm{claystone}}\;\left(\mathrm{kg}\cdot m^{-3}\right)\\ \rho_{s}\cdot c_{p,s}=p_{1}\cdot {\left(\rho \cdot c_{p}\right)}_{\mathrm{sandstone}}+p_{2}\cdot {\left(\rho \cdot c_{p}\right)}_{\mathrm{siltstone}}+p_{3}\cdot {\left(\rho \cdot c_{p}\right)}_{\mathrm{claystone}}\;\left(J\cdot m^{-3}\cdot K^{-1}\right)\\ \mathrm{for}\;\mathrm{Heat}\;\mathrm{Source}\;5 \end{array}$$

Recommended values for particular rock were used to determine the thermal conductivity coefficient $\lambda_{s}$ (W∙m^−1^∙K^−1^) for the target object Heat Source 5; intervals of possible values often vary, as stated in [11,13]. In the case of the determination of the density $\rho_{s}$ (kg∙m^−3^) and volume specific heat capacity $\rho_{s}\cdot c_{p,s}$ (J∙m^−3^∙K^−1^) for a given target object, the partial densities and partial specific heat capacities for particular types of rock were considered.

According to [15], both cases considered the percentage ratios $p_{1}$ for sandstone (main elements: quartz, feldspar), $p_{2}$ for siltstone (main elements: feldspar, quartz), and $p_{3}$ for claystone (main elements: kaolinite, illite, and montmorillonite according to [11]). [10] deals with a similar rock mixture (i.e., claystone, silty claystone, siltstone, and sandstone) in given percentage ratios used for the modeling of thermal processes focused on the deployment of the algorithm for simplified calculations of general balances for the coal mining dump and the application of this algorithm together with real data to express specific heat balances at the Hedwig mining dump.

This article contains the localized expression of the heat balance—i.e., the value of the positive heat source versus the negative values of the heat sources; see Equation (2)—in the context of “Pershing” rather than throughout the overall Hedwig mining dump, as in [22].

#### 2.1.2. Description of Technology at The Hedwig Mining Dump

To verify the mathematical model and obtain relevant data for solving problems of modeling and the prediction of thermal processes in 2012, the technology for collecting heat from places affected by a thermal process was designed, implemented, and installed. The technology for the experimentally proposed heat collector is divided into the following sections:

- The first part of the technology consists of a heat collector (“Pershing” system) serving primarily to transfer the heat from the dump (i.e., to keep cooling the dump) consisting of a cylindrical tank with a diameter of 260 mm. This tank is divided into partitions using four equal chambers whose length is 1 m. In the middle of the cylinder, there is a pipe through which the heat transfer medium flows from the secondary (cooling) part into the lower chamber (see Figure 1). Between the individual chambers, there are steel bulkheads with the holes at the edges to let the media flow upward (i.e., back to the secondary cooling circuit). Platinum resistance temperature sensors Pt100/B are installed near these holes; they represent sources of temperature data needed for a mathematical model of this technology. Additional temperature sensors are positioned at the inlet to the cylinder and the outlet from the cylinder. The whole cylinder is installed at a depth of 5 m (below ground level), at the location of the thermal process.
- The second part of the technology is composed of the secondary circuit designed to cool the heat transfer medium. In the proposed technology, the cooling process is carried out via an outdoor fan (the “Sahara”). Practically, the fluid flows from the heat exchanger using a secondary circulation pump to the radiator and then returns (already cooled) to the heat collector.

Heat transfer medium: Propylene glycol (molecular formula C_3_H_8_O_2_, also 1,2-dihydroxypropan or methylenethylenglycol): Flows from the heat collector through the primary circuit (driven by the circulating pump designed for this circuit) to the heat exchanger.

To gain knowledge about the cooling of the thermally active part of the heap, there are additional temperature sensors, a total of 24 platinum resistance temperature detectors (RTD) of Pt100/B category, installed around a collector located in two length levels and three depth levels (so-called evaluation points at depths of −2.5 m, −3.5 m, and −4.5 m; see Figure 1), whose properties, related to practical in-situ measurements and evaluations, are described in [29].

### 2.2. Development of The Numerical Mathematical Model

The description of the investigated technology plays a fairly significant role, since it allows for the consideration of other aspects of the process of creating a mathematical model in two or three dimensions:

- The first phase of the solution emphasizes the creation of a model that would be able to meet the configuration requirements to some extent (e.g., from a geometrical point of view) and the behavior (e.g., from a metrological point of view, i.e., the temperature corresponding to the real measurement) of the real temperature field at the Hedwig mining dump.
- The second phase solution emphasizes the design and implementation of the optimization problems, not only for the steady-state (i.e., stationary model) but also time-dependent job (i.e., transient model) for which the real data are available.

#### 2.2.1. Issue of Thermal Balances

According to [13], the following thermal phenomena were used to calculate the thermal balance:

- The heat needed to warm up the rock massif,
- The heat needed to warm up the coal ash,
- The heat generated or consumed due to transformations of minerals contained in the tailings,
- The heat generated by coal-burning,
- Heat loss caused by losses to the surrounding rock massif.

Let us add that the appropriate heat balance can be performed using calculations and data, based on the evaluation of the heat balance during the direct firing of the rocks. The TEBILOD program, as well as the presented COMSOL model, assumes the following premises:

- Tailings on the dump are not thermally altered (i.e., there is no conversion of the surface rocks into derivative material), and therefore the latent heat of the dump and achievable temperatures are maximum possible,
- No thermally altered coal mass from the past is considered,
- No heat losses are considered.

The TEBILOD program operates with the amount of burned carbon contained in the tailings $w_{\mathrm{coal}}$ (kg∙kg^−1^) and with a function describing the maximum achievable Celsius temperature $\vartheta_{\mathrm{desired},\max}=f\left(w_{\mathrm{coal}}\right)$ (°C) (see [13,22]). In contrast, the COMSOL model works with the values of the heat sources for the heat transfer medium ${\dot{Q}}_{1}$ to ${\dot{Q}}_{4}$ (in Watts) and the surrounding waste rock ${\dot{Q}}_{\mathrm{source}}$ (in Watts), which depends on the temperatures (°C) in the chambers and around the “Pershing” system. The effect of the amount of burnt coal in the tailings, in this case, is not considered.

If the stationary task in the steady-state is considered, it must follow, according to [30,31], that the sum of the negative heat sources is equal to the sum of the positive heat sources. It is then possible to state the balance equation of heat sources for the mathematical model (steady-state):

(2) $$\begin{array}{c} \begin{array}{ccc} {\dot{Q}}_{\mathrm{source}}+\overset{4}{\underset{i=1}{\sum }}{\dot{Q}}_{i}=0 & & \left(W\right) \end{array}\\ \begin{array}{ccc} \underset{\begin{array}{c} \mathrm{negative}\;\mathrm{values}\\ \mathrm{of}\;\mathrm{heat}\;\mathrm{sources} \end{array}}{\underbrace{-\overset{4}{\underset{m=1}{\sum }}{\dot{Q}}_{m}}}=\underset{\begin{array}{c} \mathrm{positive}\;\mathrm{value}\\ \mathrm{of}\;\mathrm{heat}\;\mathrm{source} \end{array}}{\underbrace{{\dot{Q}}_{\mathrm{source}}}} & & \left(W\right) \end{array} \end{array}$$

Based on Equation (2), it is possible to make a rough guess of ${\dot{Q}}_{\mathrm{source}}$ and thus get starting settings of the optimization problem.

According to [13], it is possible to guess the amount of energy released during the burning of coal matter. The calculation of heat transfer in a burning heap includes the type of the coal and its specific combustion heat, moisture, ash content, the chemical and mineralogical composition of the rock [see Equation (1) and Table 2], the thermal conductivity of the tailing, and the dump volume (see also Table 1). These parameters are usually unknown, so it is necessary to set up their *ad-hoc* estimates. In practice, the reliable information about the start and end of the burning process is not usually available [13]; furthermore, the current state of knowledge of the chemistry of the main types of rocks and their corresponding mineralogical composition (typically carboniferous rocks) is not entirely satisfactory. If a chemical analysis of the selected types of rocks is only at one’s disposal, there is often a lack of exact identification and quantification of the mineral composition of rocks (especially for clay materials or carbonates).

#### 2.2.2. Input Parameters of The Mathematical Model

In a real vertical borehole heat exchanger, according to [23], at least one collector loop (i.e., one input to the loop and one output of the loop; configuration 1-U) is available, through which the heat transfer medium flows and takes the heat from the surrounding rock environment. In other words, the concept of this model assumes that the surrounding rock environment represents a source of heat (a positive value) and that the flowing transfer medium is the heat consumer (negative value); see also Equation (2). Such a concept of the model assumes the following simplifications:

- Collector reduction: Only one tube with a given initial temperature (at the collector inlet/outlet boundary) at a given depth,
- The geometry of the collector: The collector (“Pershing” system) is vertical along its length,
- The character of the rock massif: Within the frame of the modeled domain, it is considered as a homogenous environment in terms of the percentage composition of the given rocks, see Equation (1).

#### 2.2.3. Conceptual Design of The Mathematical Model

From a geometric point of view, it is obvious that the ranges of heat sources have their borders on so-called domains (see Table 3). If the geometry of the model allows the use of so-called axial symmetry, the number of the domains drops twice (see also Figure 1); the same thing also holds in the case of boundaries with the predefined boundary condition of the 1st to the 3rd kinds.

Based on the values of the heat sources, it is possible to define the following problems to be solved:

- Interpretation of Qsource parameter in the 2D model: If the volume heat source is considered, three dimensions are needed (i.e., length, width, and height); the sectional view offers two dimensions only (length and width)—it is necessary to adjust the third dimension,
- Interpretation of the unit of Qsource parameter in the 2D model: In case the value of the heat source is given in Watts (W), we want it to be expressed into Watts per cubic meter (W∙m^−3^),
- Interpretation of Qsource parameter in the 3D model: If the volume heat source is considered, three dimensions are needed (i.e., length, width, and height); the 3D view offers all three dimensions, and the problem can be turned into the calculation of the volume of a given domain,
- Interpretation of the unit of Qsource parameter in the 3D model: In case the value of the heat source is given in Watts (W), we want it to be expressed into Watts per cubic meter (W∙m^−3^) based on the volume value in the 3D model.

Based on the geometry of the heat collector, the volumes of particular domains in the 2D model, and consequently also in the 3D model, are calculated. The axial symmetry of the model domain is considered (see Figure 1).

Based on the properties of the generalized conceptual model, the following forms of equations and parameters related to particular domains are considered:

- Heat transfer in solids: In the model, this is related to the target object Heat Source 5 (i.e., solid-phase—waste rocks; positive heat source) with the material parameters given by Table 2.
- Thermal isolation: In the model, this is related to the transitional sand layer (between waste rocks and steel tube if the collector is sand), with the following material parameters: $\lambda_{\mathrm{sand}}=0.95\;W\cdot m^{-1}\cdot K^{-1}$, $\rho_{\mathrm{sand}}=1750\;\mathrm{kg}\cdot m^{-3}$, and $c_{p,\mathrm{sand}}=960\;J\cdot {\mathrm{kg}}^{-1}\cdot K^{-1}$,
- Heat sources: In the model, this is related to the target objects Heat Source 1 to Heat Source 4 (i.e., liquid phase: propylene glycol; negative heat sources), with the material parameters related to pro propylene glycol given by Equation (10),
- Highly conductive layer: In the model, this is related to the steel tube (outer cover of the “Pershing” system; structured steel) related to Equation (8) and with the following material parameters: $\lambda_{\mathrm{steel}}=44.50\;W\cdot m^{-1}\cdot K^{-1}$, $\rho_{\mathrm{steel}}=7850\;\mathrm{kg}\cdot m^{-3}$, and $c_{p,\mathrm{steel}}=475\;J\cdot {\mathrm{kg}}^{-1}\cdot K^{-1}$.

#### 2.2.4. Control Equations of The Mathematical Model

##### Control Equations for A Rock Environment

The general form of the resulting inhomogeneous PDE (with consideration of the internal heat source *Q* in the Cartesian coordinate system) for heat transfer in solids is given by the following relations in the form of a differential operator (in this case, the Hamiltonian operator nabla, i.e., ∇ m^−1^), as stated in [12,19]:

For a heat source with the overall heat power (in Watts; parameter Ptot; 3D model, transient job):

(3) $$\begin{array}{cc} PDE: & \rho \cdot c_{p}\cdot \frac{\partial T}{\partial t}+\rho \cdot c_{p}\cdot u\cdot \nabla T=\nabla \cdot \left(\lambda \cdot \nabla T\right)+Q\;\left(W\cdot m^{-3}\right)\\ & Q\equiv {\dot{Q}}_{V,\;s}=\frac{P_{\mathrm{tot}}}{V_{s}}\;\left(W\cdot m^{-3}\right)\\ & u\equiv u_{s}=[u_{1,s},u_{2,s},u_{3,s}]\equiv 0\;\left(m\cdot s^{-1}\right)\\ & x=\left[x,y,z\right]\in {\mathbb{R}}^{3}\;\left(m\right)\\ & \rho \cdot c_{p}\equiv \rho_{s}\cdot c_{p,s}=\mathrm{const}.\;\left(J\cdot m^{-3}\cdot K^{-1}\right)\\ & \lambda \equiv \lambda_{s}=\lambda_{x}=\lambda_{y}=\lambda_{z}=\mathrm{const}.\;\left(W\cdot m^{-1}\cdot K^{-1}\right)\\ & T\equiv T_{s}\left(x,t\right)=T_{s}\left(x,y,z,t\right)\;\left(K\right)\\ & Q\equiv Q_{s}\left(x,t\right)=Q_{s}\left(x,y,z,t\right)\;\left(W\cdot m^{-3}\right)\\ & t\in [0,+\infty )\;\left(s\right) \end{array}$$

For a general volume heat source (in W∙m^−3^; 3D model, transient job):

(4) $$\begin{array}{cc} PDE: & \rho \cdot c_{p}\cdot \frac{\partial T}{\partial t}+\rho \cdot c_{p}\cdot u\cdot \nabla T=\nabla \cdot \left(\lambda \cdot \nabla T\right)+Q\;\left(W\cdot m^{-3}\right)\\ & Q\equiv {\dot{Q}}_{V,s}\;\left(W\cdot m^{-3}\right)\\ & u\equiv u_{s}=\left[u_{1,s},u_{2,s},u_{3,s}\right]\equiv 0\;\left(m\cdot s^{-1}\right)\\ & x=\left[x,y,z\right]\in {\mathbb{R}}^{3}\;\left(m\right)\\ & \rho \cdot c_{p}\equiv \rho_{s}\cdot c_{p,s}=\mathrm{const}.\;\left(J\cdot m^{-3}\cdot K^{-1}\right)\\ & \lambda \equiv \lambda_{s}=\lambda_{x}=\lambda_{y}=\lambda_{z}=\mathrm{const}.\;\left(W\cdot m^{-1}\cdot K^{-1}\right)\\ & T\equiv T_{s}\left(x,t\right)=T_{s}\left(x,y,z,t\right)\;\left(K\right)\\ & Q\equiv Q_{s}\left(x,t\right)=Q_{s}\left(x,y,z,t\right)\;\left(W\cdot m^{-3}\right)\\ & t\in [0,+\infty )\;\left(s\right) \end{array}$$

For a heat source with an overall heat power (in Watts; parameter Ptot; 3D model, stationary job):

(5) $$\begin{array}{cc} PDE: & \rho \cdot c_{p}\cdot u\cdot \nabla T=\nabla \cdot \left(\lambda \cdot \nabla T\right)+Q\;\left(W\cdot m^{-3}\right)\\ & Q\equiv {\dot{Q}}_{V,s}=\frac{P_{\mathrm{tot}}}{V_{s}}\;\left(W\cdot m^{-3}\right)\\ & u\equiv u_{s}=\left[u_{1,s},u_{2,s},u_{3,s}\right]\equiv 0\;\left(m\cdot s^{-1}\right)\\ & x=\left[x,y,z\right]\in {\mathbb{R}}^{3}\;\left(m\right)\\ & \rho \cdot c_{p}\equiv \rho_{s}\cdot c_{p,s}=\mathrm{const}.\;\left(J\cdot m^{-3}\cdot K^{-1}\right)\\ & \lambda \equiv \lambda_{s}=\lambda_{x}=\lambda_{y}=\lambda_{z}=\mathrm{const}.\;\left(W\cdot m^{-1}\cdot K^{-1}\right)\\ & T\equiv T_{s}\left(x\right)=T_{s}\left(x,y,z\right)\;\left(K\right)\\ & Q\equiv Q_{s}\left(x,t\right)=Q_{s}\left(x,y,z,t\right)\;\left(W\cdot m^{-3}\right)\\ & t\to +\infty \;\left(s\right) \end{array}$$

For a general volume heat source (in W∙m^−3^; 3D model, stationary job):

(6) $$\begin{array}{ccc} Q\equiv {\dot{Q}}_{V,s} & & \left(W\cdot m^{-3}\right) \end{array}$$

Let us add that the above-mentioned Equations (3) to (6) consider the constant isotropic thermal conductivity coefficient, i.e., $\lambda \equiv \lambda_{s}=\lambda_{x}=\lambda_{y}=\lambda_{z}=\mathrm{const}.$ (W∙m^−1^∙K^−1^; see also Table 2), resp. constant isotropic heat diffusivity, i.e., $a\equiv a_{s}=a_{x}=a_{y}=a_{z}=\mathrm{const}.$ (m^2^∙s^−1^; see also Table 2).

Formal adjustments of Equations (3)–(6) lead to known forms for inhomogeneous partial differential equations for heat conduction in solids; while related to Equation (3), it is possible to write down the following relations for the Cartesian coordinate system (see also [19]):

(7) PDE:ρ·cp·(∂T∂t+u·∇T)=∇·(λ·∇T)+Q˙V (W·m−3)  ρs·cp,s·{∂Ts∂t+us·grad(Ts)}=λs·div(∇Ts)+∇Ts·grad(λs)+Q˙V,s ρs·cp,s·(∂Ts∂t+us·∇Ts)=λs·∇·∇Ts+∇Ts·∇λs+Q˙V,s ρs·cp,s·(∂Ts∂t+us·∇Ts)⏟left side in (W·m−3)=λs·ΔTs+∇Ts·∇λs+Q˙V,s⏟right side in (W·m−3)   x=[x,y,z]∈ℝ3 (m) u≡us=[u1,s,u2,s,u3,s]=0 (m·s−1) T≡Ts=Ts(x,t)=Ts(x,y,z,t) (K) t∈[0,+∞) (s)

##### Control Equations for A Steel Tube

In the “Pershing” system, all negative heat sources (i.e., domains 3,4 and 7,5 and 8,6 and 9; see Table 3) are delimited by a steel tube, forming the interface between the propylene glycol as a heat transfer fluid and sand as a transition layer between the steel pipe and the surrounding rock environment. This tube is, in the model, implemented in the Trubka hranice object comprising the boundary interfaces 7 and 29 (domain 3), 9 and 31 (for domains 4 and 7), 11 and 32 (for domains 5 and 8), and 13 and 33 (for domains 6 and 9). In this model, it is the only explicitly defined boundary condition.

The generalized form of the resulting inhomogeneous partial differential equation for heat transfer in highly conductive layers (HCL)—in this case in the steel tube (i.e., “Pershing” system)—is given by the relations in the form of differential operators (in this case, the Hamiltonian operator nabla of the highly conductive layer; i.e., $\nabla_{\mathrm{HCL}}$ in m^−1^) with the Neumann boundary condition (wherein the symbols, according to V≡Ω, denote a particular domain, and ∂V≡∂Ω or S≡Γ signifies a particular boundary, on which there is the prescribed specific boundary condition); then (for a Cartesian coordinate system; see also [19]), the following relation can be stated:

(8)  −q″tub=q″∂V−q″V+dtub·Q˙tub PDE:−q″tub=dtub·ρtub·cp,tub·∂Ttub∂t+∇HCL·(−dtub·κtub·∇HCLTtub) (W·m−2)  −q″tub=dtub·ρtub·cp,tub·∂Ttub∂t−dtub·λtub·ΔHCLTtub BC:−n·q″=−dtub·ρtub·cp,tub·∂Ttub∂t−∇HCL·(−dtub·κtub·∇HCLTtub) on ∂V

Let us add that the boundary condition in Equation (8) is generalized.

The boundary 17 (domain 3; implementation in the target object Heat Source 1), in the case of the 2D model, contains the initial condition formulated in the form of the initial temperature of the heat transfer media (default in Kelvins); thus, for the Cartesian coordinate system, it is as follows (see also Figure 1):

(9) t=t0:Tf(x,t0)=Tf(x)in V¯3,17=V3∪S17x∈S17 t=0:Tf=Tf(x)≡T0=293.15 Kt=0:ϑf=ϑf(x)≡ϑ0=20.00 °C

#### 2.2.5. Output Parameters of The Mathematical Model

##### Design of An Optimization Task

For the design of the optimization problem, it is necessary to consider the following issues:

- The dimension of the model: 2D model (see Figure 1), alternatively 3D model,
- Type of the job: Stationary job,
- Measured real data: From 18. 3. 2013 (11:30:00) to 28. 3. 2013 (23:30:00),
- Simulated data from the model: 2D model and 3D model (yz-plane or xyz-cut plane and given evaluation point),
- Evaluation points: Point B (−2.5 m), point C (−3.5 m), point D (−4.5 m),
- Evaluation points (in chambers): Point HK (−2.0 m), point PK (−3.0 m), point DK (−4.0 m),
- Inputs to optimization task: Quantities ${\dot{Q}}_{1}$ (Watts), ${\dot{Q}}_{2}$ (Watts), ${\dot{Q}}_{3}$ (Watts), ${\dot{Q}}_{4}$ (Watts),
- Optimized quantity: Quantity ${\dot{Q}}_{\mathrm{source},\mathrm{est}.}$ (Watts),
- Optimization criteria: Absolute value of the difference between simulated data and real measured data,
- Data exchange: With the use of the LiveLink for MATLAB (see Figure 2),
- The output from optimization task: Minimization of optimization criteria function for ${\dot{Q}}_{\mathrm{source},\mathrm{est}.}$

Let us add that the block scheme of the stationary jobs (see Figure 2) can, after minor modifications, also be applied for transient jobs. Modifications would be related to the signal $T_{\mathrm{data}}$ (Kelvins) and the objective function, $J\left[T_{\mathrm{model}},\;T_{\mathrm{data}}\right]$ (Kelvins).

##### Definition of Equations for Inputs of The Optimization Task

The basic state equation for this optimization task is represented by the temperature, which depends on available real measured data, i.e., the media temperature ($T_{f_{\mathrm{IN}}}$ and $T_{f_{\mathrm{OUT}}}$; Kelvins), the temperature of the media in three chambers ($T_{\mathrm{DK}}$, $T_{\mathrm{PK}}$, $T_{\mathrm{HK}}$; K), and the temperature at three evaluation points ($T_{B}$, $T_{C}$, $T_{D}$; K). Within the preprocessing phase (see Figure 2), the mutual relations between these data and the input quantities entering into optimization are as follows:

(10) Q˙m≡Q˙fm=Kfm·ΔTfm Q˙fm=ρfm·cp,fm⏟known·QV,fm·ΔTfm⏟measured(W) ρfm=1036.000 (kg·m−3)cp,fm=3370.374 (J·kg−1·K−1)QV,fm=1.500×10−3 (m3·s−1)Kfm=5237.561 (W·K−1) for m={1,2,3,4} at Q˙1>Q˙2>Q˙3>Q˙4 if Q˙1<0Q˙2<0Q˙3<0Q˙4<0

After expression for the heat source ${\dot{Q}}_{1}$ (in Watts; temperature difference between lower chamber and inlet media temperature):

(11) $$\begin{array}{ccc} \begin{array}{c} {\dot{Q}}_{1}\equiv {\dot{Q}}_{f_{1}}=K_{f_{1}}\cdot \Delta T_{f_{1}}=\rho_{f_{1}}\cdot c_{{p,f}_{1}}\cdot Q_{{v,f}_{1}}\cdot \left(T_{\mathrm{DK}}-T_{f_{\mathrm{IN}}}\right)\\ {\dot{Q}}_{f_{1}}=5237.561\cdot \left(T_{\mathrm{DK}}-T_{f_{\mathrm{IN}}}\right) \end{array} & & \left(W\right) \end{array}$$

After expression for the heat source ${\dot{Q}}_{2}$ (in Watts; temperature difference between the middle chamber and lower chamber):

(12) $$\begin{array}{ccc} \begin{array}{c} {\dot{Q}}_{2}\equiv {\dot{Q}}_{f_{2}}=K_{f_{2}}\cdot \Delta T_{f_{2}}=\rho_{f_{2}}\cdot c_{{p,f}_{2}}\cdot Q_{{v,f}_{2}}\cdot \left(T_{\mathrm{PK}}-T_{\mathrm{DK}}\right)\\ Q_{f_{2}}=5237.561\cdot \left(T_{\mathrm{PK}}-T_{\mathrm{DK}}\right) \end{array} & & \left(W\right) \end{array}$$

After expression for the heat source ${\dot{Q}}_{3}$ (in Watts; temperature difference between the upper chamber and middle chamber):

(13) $$\begin{array}{ccc} \begin{array}{c} {\dot{Q}}_{3}\equiv {\dot{Q}}_{f_{3}}=K_{f_{3}}\cdot \Delta T_{f_{3}}=\rho_{f_{3}}\cdot c_{{p,f}_{3}}\cdot Q_{{v,f}_{3}}\cdot \left(T_{\mathrm{HK}}-T_{\mathrm{PK}}\right)\\ {\dot{Q}}_{f_{1}}=5237.561\cdot \left(T_{\mathrm{HK}}-T_{\mathrm{PK}}\right) \end{array} & & \left(W\right) \end{array}$$

After expression for the heat source ${\dot{Q}}_{4}$ (in Watts; temperature difference between output media and upper chamber):

(14) $$\begin{array}{ccc} \begin{array}{c} {\dot{Q}}_{4}\equiv {\dot{Q}}_{f_{4}}=K_{f_{4}}\cdot \Delta T_{f_{4}}=\rho_{f_{4}}\cdot c_{{p,f}_{4}}\cdot Q_{{v,f}_{4}}\cdot \left(T_{f_{\mathrm{OUT}}}-T_{\mathrm{HK}}\right)\\ {\dot{Q}}_{f_{4}}=5237.561\cdot \left(T_{f_{\mathrm{OUT}}}-T_{\mathrm{HK}}\right) \end{array} & & \left(W\right) \end{array}$$

The above-mentioned properties are valid for both types of solved problems (i.e., stationary and transient); for the selected form of the optimization criteria, they are as follows:

Generally, for a stationary job and N value of the signal $x_{1}\equiv T_{\mathrm{model}}$ and $x_{2}\equiv T_{\mathrm{real}}$ (K):

(15) $$\begin{array}{cc} t\in [t_{0},t_{1}):\;J\left[x_{1}\left(t\right),x_{2}\left(t\right)\right]=\min\left|x_{1}\left(t\right)-x_{2}\left(t\right)\right|\\ \mathrm{for}\;t\to +\infty :\;x_{2}\left(t\right)=\mathrm{const}.\\ ⇓\\ \begin{array}{c} J\left[x_{1}\left(i\right),x_{2}\left(i\right)\right]=\min\left|x_{1}\left(t\right)-x_{2}\right|\\ J\left[T_{\mathrm{model}},T_{\mathrm{real}}\right]=\min\left|T_{\mathrm{model}}\left[i\right]-T_{\mathrm{real}}\right| \end{array} & \left(K\right)\\ \mathrm{for}\;i=\{1,2,\ldots ,M\} \end{array}$$

For a particular stationary job in this paper and N values of the signal $x_{1}\equiv T_{\mathrm{model}}$ a $x_{2}\equiv T_{\mathrm{data}}$ (K):

(16) $$\begin{array}{cc} t\in [t_{0},t_{1}):\;J\left[x_{1}\left(t\right),x_{2}\left(t\right)\right]=\min\left|x_{1}\left(t\right)-x_{2}\left(t\right)\right|\\ ⇓\\ J\left[T_{\mathrm{model}},T_{\mathrm{data}}\right]=\min\left|T_{\mathrm{model}}\left[i\right]-\bar{T_{\mathrm{data}}\left[i\right]}\right|\\ \begin{array}{c} J\left[T_{\mathrm{pev},2_{\left(B=-2.5\;m\right)}},T_{B}\equiv \bar{T_{B}}\right]=\min\left|T_{\mathrm{pev},2_{\left(B=-2.5\;m\right)}}\left[i\right]-\bar{T_{B}\left[i\right]}\right|\\ J\left[T_{\mathrm{pev},2_{\left(C=-3.5\;m\right)}},T_{C}\equiv \bar{T_{C}}\right]=\min\left|T_{\mathrm{pev},2_{\left(C=-2.5\;m\right)}}\left[i\right]-\bar{T_{C}\left[i\right]}\right| \end{array} & \left(K\right)\\ J\left[T_{\mathrm{pev},2_{\left(D=-2.5\;m\right)}},T_{D}\equiv \bar{T_{D}}\right]=\min\left|T_{\mathrm{pev},2_{\left(D=-2.5\;m\right)}}\left[i\right]-\bar{T_{D}\left[i\right]}\right|\\ \mathrm{for}\;i=\left\{1,2,\ldots ,N\right\} \end{array}$$

which leads to the form of the objective function based on the measured thermal profile Equation (17):

(17) $$J\left[T_{\mathrm{pev},2_{\left(B=-2.5\;m\right)}},T_{B}\equiv \bar{T_{B}}\right]+J\left[T_{\mathrm{pev},3_{\left(C=-3.5\;m\right)}},T_{C}\equiv \bar{T_{C}}\right]+J\left[T_{\mathrm{pev},4_{\left(D=-4.5\;m\right)}},T_{D}\equiv \bar{T_{D}}\right]$$

From Equations (15) and (16), it is obvious that the signal $T_{\mathrm{data}}$ (Kelvins) can be chosen as the real measured signals $T_{B}\left(t\right)$, $T_{C}\left(t\right)$, $T_{D}\left(t\right)$ (Kelvins), or their discretized forms $T_{B}\left[i\right]$, $T_{C}\left[i\right]$, $T_{D}\left[i\right]$ at the evaluation points B, C, and D. Equations (15) and (16) express the form of the optimization criteria for each evaluation point (thus speaking of so-called *point optimization*), while Equation (17) represents the optimization criteria for more evaluation points at the same time (thus speaking of so-called thermal profile-based optimization).

The output of the optimization task is represented by the estimated value of ${\dot{Q}}_{\mathrm{source}}$, i.e., ${\dot{Q}}_{\mathrm{source},\mathrm{est}.}$ (Watts), at a given temperature (at the evaluation point). On the other hand, the corrected estimation value of ${\dot{Q}}_{\mathrm{source}}$, i.e., ${\dot{Q}}_{\mathrm{source},\mathrm{corr}.}$ (Watts), is calculated, at the particular evaluation point, from general graphical dependence $\dot{Q}=f\left(T\right)$ (Watts), wherein in terms of coordinates the relations are: $P_{\mathrm{corr}.,B}=\left[T=\bar{T_{B}};\dot{Q}={\dot{Q}}_{\mathrm{source},\mathrm{corr}._{B}}\right]$, $P_{\mathrm{corr}.,C}=\left[T=\bar{T_{C}};\dot{Q}={\dot{Q}}_{\mathrm{source},\mathrm{corr}._{C}}\right]$, and $P_{\mathrm{corr}.,D}=\left[T=\bar{T_{D}};\dot{Q}={\dot{Q}}_{\mathrm{source},\mathrm{corr}._{D}}\right]$. The interpolation of the dependence $\dot{Q}=f\left(T\right)$ is not generally linear.

## 3. Results

### 3.1. Determining The Value of ${\dot{Q}}_{\mathrm{source}}$

The values of the negative heat sources ${\dot{Q}}_{1}$ to ${\dot{Q}}_{4}$ (W) are evaluated based on Equations (10) to (14); see also Figure 2. Their representation come either from measured data in chambers (i.e., sources ${\dot{Q}}_{2}$ and ${\dot{Q}}_{3}$; see Figure 1) or from estimations based on the temperature difference between the collector’s inlet and outlet (i.e., temperatures $T_{f\mathrm{IN}}$ and $T_{f\mathrm{OUT}}$; Kelvins) in the “Pershing” system (i.e., sources ${\dot{Q}}_{1}$ and ${\dot{Q}}_{4}$; Watts).

On the other hand, the value of the positive heat source ${\dot{Q}}_{\mathrm{source},\mathrm{opt}.}$ (Watts) is calculated by a static optimization job (see Equation (15) or (16), and also Figure 2), based on the search of the local minimum in a given range (vector L, Matlab function fmincon) in which the optimized value is looked for.

#### 3.1.1. The Case Study I: Stationary Model (Reference Values)

This study introduces a solution considering the reference (or nominal) value of these various heat sources for the implementation of a sensitivity analysis. Data in Table 4 are valid for the period between 24. 3. 2013 (5:40) and 28. 3. 2013 (23:30), i.e., 113.83 h, and processes within the solution of the stationary job.

The values of heat sources ${\dot{Q}}_{2}$ and ${\dot{Q}}_{3}$ reflect the temperature growth presented in the data, while $\bar{\vartheta_{\mathrm{DK}}}=6.1765\;{}^{\circ}C$, $\bar{\vartheta_{\mathrm{PK}}}=6.1742\;{}^{\circ}C$, and $\bar{\vartheta_{\mathrm{HK}}}=6.1642\;{}^{\circ}C$. The temperatures in the chambers (see Figure 2) were averaged from 684 samples (at a sampling period $T_{S}=10\;\min=600\;s$). It is clear that $\bar{T_{\mathrm{DK}}}=\bar{\vartheta_{\mathrm{DK}}}+273.15$ (Kelvins), $\bar{T_{\mathrm{PK}}}=\bar{\vartheta_{\mathrm{PK}}}+273.15$ (Kelvins), and $\bar{T_{\mathrm{HK}}}=\bar{\vartheta_{\mathrm{HK}}}+273.15$ (Kelvins).

Particular temperatures at evaluation points are as follows: $\bar{\vartheta_{B}}=20.4604\;{}^{\circ}C$, $\bar{\vartheta_{C}}=33.1705\;{}^{\circ}C$, and $\bar{\vartheta_{D}}=41.8420\;{}^{\circ}C$. Temperatures at particular evaluation points (see Figure 2) were also averaged from 684 samples (at a sampling period $T_{S}=10\;\min=600\;s$). It is clear that $\bar{T_{B}}=\bar{\vartheta_{B}}+273.15$ (Kelvins), $\bar{T_{C}}=\bar{\vartheta_{C}}+273.15$ (Kelvins), and $\bar{T_{D}}=\bar{\vartheta_{D}}+273.15$ (Kelvins).

**Figure 3:**
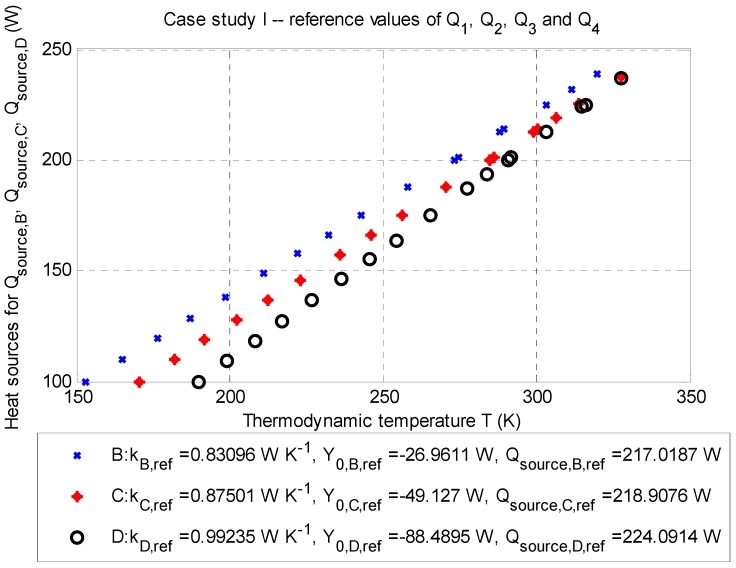
The case study I: Q-T diagram for evaluation points B (depth: −2.5 m), C (depth: −3.5 m), and D (depth: −4.5 m) with determined approximated parameters of the line and corrected values of the positive heat sources ${\dot{Q}}_{source,corr._{B}}$, ${\dot{Q}}_{\mathrm{source},\mathrm{corr}._{C}}$, and ${\dot{Q}}_{\mathrm{source},\mathrm{corr}._{D}}$ (Watts) for particular thermodynamic temperatures $\bar{T_{B}}$, $\bar{T_{C}}$, and $\bar{T_{D}}$ (Kelvins); stationary job, 2D model.

For comparison purposes, the data in Table 5 and Figure 3 are valid for the same period as the data in Table 4.

The balance of the heat source, according to Equation (2) for the point optimization, is as follows: $\sum_{m=1}^{4}{\dot{Q}}_{m,\mathrm{CS}\;I}=-202.6938\;W$ (for negative heat sources) and $\bar{{\dot{Q}}_{\mathrm{source},\mathrm{corr}.}}=+220.0059\;W$ (for positive heat source).

#### 3.1.2. Case Study II: Stationary Model (±10% of Reference V of ${\dot{Q}}_{1}$ and ${\dot{Q}}_{4}$)

**Figure 4:**
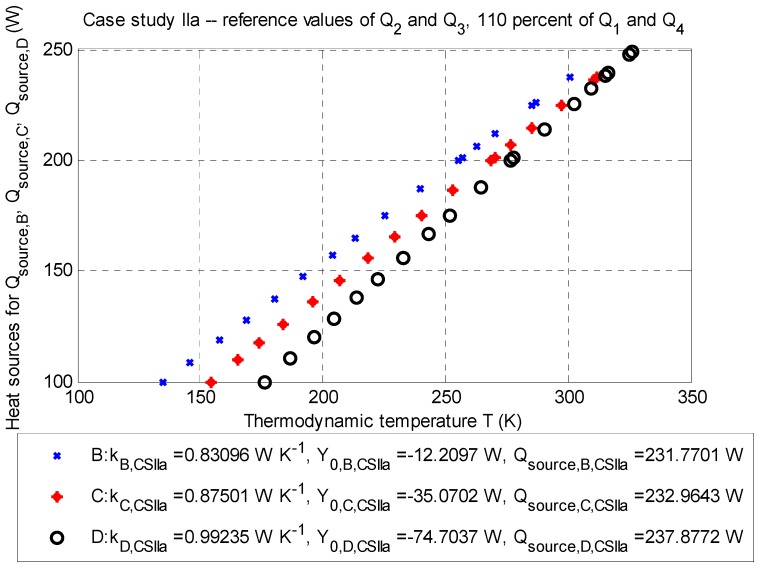
Case study IIa: Q-T diagram for evaluation points B (depth: −2.5 m), C (depth: −3.5 m), and D (depth: −4.5 m) with determined approximated parameters of the line and corrected values of the positive heat sources ${\dot{Q}}_{\mathrm{source},\mathrm{corr}._{B}}$, ${\dot{Q}}_{\mathrm{source},\mathrm{corr}._{C}}$ and ${\dot{Q}}_{\mathrm{source},\mathrm{corr}._{D}}$ (Watts) for particular thermodynamic temperatures $\bar{T_{B}}$, $\bar{T_{C},}$ and $\bar{T_{D}}$ (K); stationary job, 2D model.

**Figure 5:**
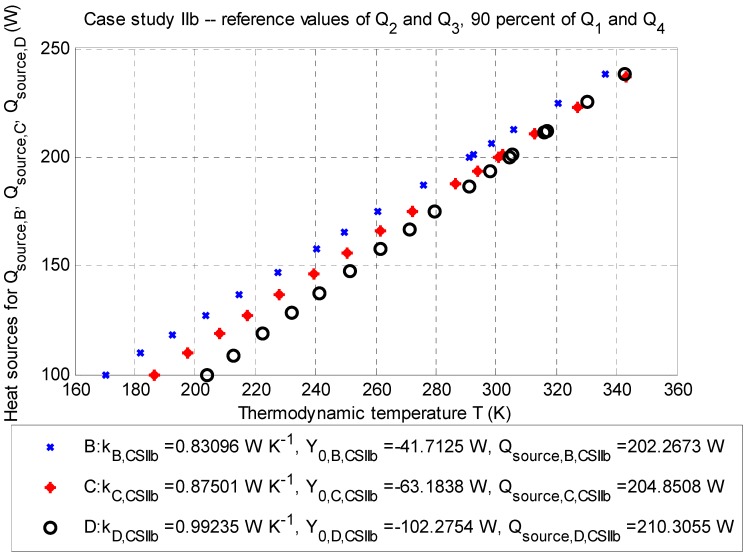
Case study IIb: Q-T diagram for evaluation points B (depth: −2.5 m), C (depth: −3.5 m), and D (depth: −4.5 m) with the determined approximated parameters of the line and corrected values of the positive heat sources ${\dot{Q}}_{\mathrm{source},\mathrm{corr}._{B}}$, ${\dot{Q}}_{\mathrm{source},\mathrm{corr}._{C}}$, and ${\dot{Q}}_{\mathrm{source},\mathrm{corr}._{D}}$ (Watts) for particular thermodynamic temperatures $\bar{T_{B}}$, $\bar{T_{C}}$, and $\bar{T_{D}}$ (K); stationary job, 2D model.

This case study investigates the influence of the value of the heat sources ${\dot{Q}}_{1}$ and ${\dot{Q}}_{4}$ (Watts), given by the estimation of temperature differences in Equations (11) and (14). The results of this case studies are summarized in Table 6, Table 7, Table 8 and Table 9. Data are plotted in Figure 4 and Figure 5.

Case study IIa: The influence of a +10% increase of reference values.

The balance of the heat source is according to Equation (2) for the point optimization, as follows: $\sum_{m=1}^{4}{\dot{Q}}_{m,\mathrm{CS}\;\mathrm{IIa}}=-216.5210\;W$ (for negative heat sources) and $\bar{{\dot{Q}}_{\mathrm{source},\mathrm{corr}.}}=+234.2039\;W$ (positive heat sources).

##### Case Study IIb: The Influence of −10% of Reference Values

The balance of the heat source is according to Equation (2) for the point optimization, as follows: $\sum_{m=1}^{4}{\dot{Q}}_{m,\mathrm{CS}\;\mathrm{IIb}}=-188.8666\;W$ (for negative heat sources) and $\bar{{\dot{Q}}_{\mathrm{source},\mathrm{corr}.}}=+205.8079\;W$ (for positive heat source).

#### 3.1.3. Case Study III: Stationary Model (±10% of Reference Values of ${\dot{Q}}_{2}$ and ${\dot{Q}}_{3}$)

**Figure 6:**
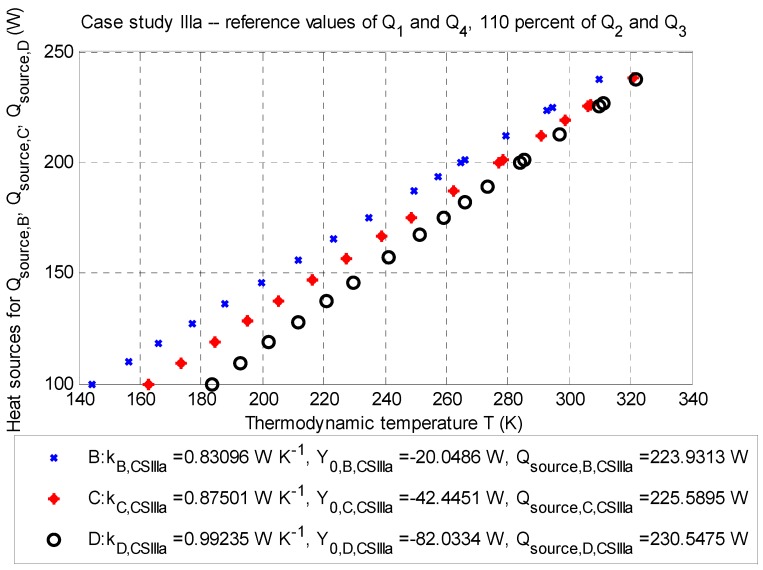
Case study IIIa—Q-T diagram for evaluation points B (depth: −2.5 m), C (depth: −3.5 m), and D (depth: −4.5 m) with the determined approximated parameters of the line and corrected values of the positive heat sources ${\dot{Q}}_{\mathrm{source},\mathrm{corr}._{B}}$, ${\dot{Q}}_{\mathrm{source},\mathrm{corr}._{C}}$, and ${\dot{Q}}_{\mathrm{source},\mathrm{corr}._{D}}$ (Watts) for particular thermodynamic temperatures $\bar{T_{B}}$, $\bar{T_{C},}$ and $\bar{T_{D}}$ (Kelvins); stationary job, 2D model.

**Figure 7:**
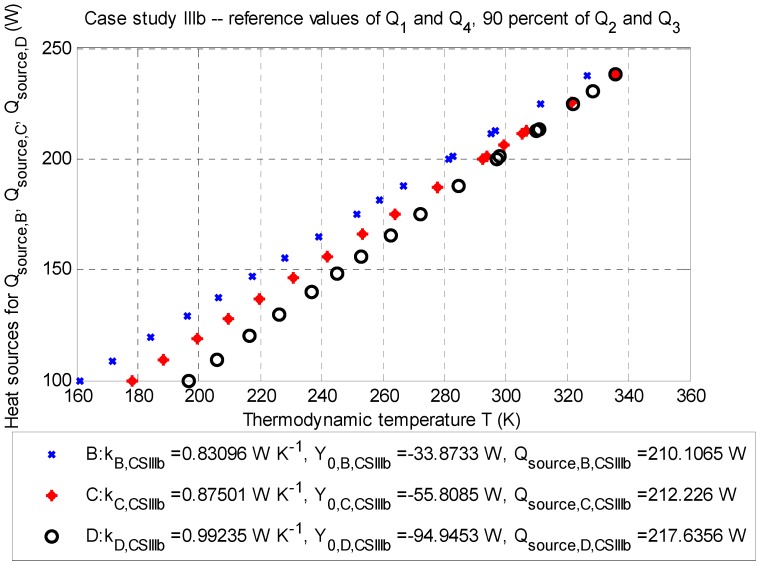
Case study IIIb: Q-T diagram for evaluation points B (depth: −2.5 m), C (depth: −3.5 m), and D (depth: −4.5 m) with the determined approximated parameters of the line and corrected values of the positive heat sources ${\dot{Q}}_{\mathrm{source},B}$, ${\dot{Q}}_{\mathrm{source},C}$, and ${\dot{Q}}_{\mathrm{source},D}$ (Watts) for particular thermodynamic temperatures $\bar{T_{B}}$, $\bar{T_{C}}$, and $\bar{T_{D}}$ (Kelvins); stationary job, 2D model.

This case study investigates the influence of the value of the heat sources ${\dot{Q}}_{2}$ and ${\dot{Q}}_{3}$ (Watts), given by estimation of temperature differences in Equations (12) and (13). The results of this case studies are summarized in Table 10, Table 11, Table 12 and Table 13. Data are plotted in Figure 6 and Figure 7.

##### Case Study IIIa: The Influence of +10% of Reference Values

The balance of the heat source is according to Equation (2) for the point optimization, as follows: $\sum_{m=1}^{4}{\dot{Q}}_{m,\mathrm{CS}\;\mathrm{IIIa}}=-209.1363\;W$ (for negative heat sources) and $\bar{{\dot{Q}}_{\mathrm{source},\mathrm{corr}.}}=+226.6895\;W$ (for positive heat source).

##### Case Study IIIb: The Influence of −10% of Reference Values

The balance of the heat source is according to Equation (2) for the point optimization, as follows: $\sum_{m=1}^{4}{\dot{Q}}_{m,\mathrm{CS}\;\mathrm{IIIb}}=-196.2516\;W$ (for negative heat sources) and $\bar{{\dot{Q}}_{\mathrm{source},\mathrm{corr}.}}=+213.3227\;W$ (for positive heat source).

### 3.2. Validating The Value of ${\dot{Q}}_{\mathrm{source}}$

All the above case studies presented not only estimates of the variable, ${\dot{Q}}_{\mathrm{source},\mathrm{est}.}$ (W), but also the corrected value, ${\dot{Q}}_{\mathrm{source},\mathrm{corr}.}$ (W), either according to a known value of the thermodynamic temperature in the respective evaluation point (see Figure 1 and Figure 2), or depending on the temperature profile of the measured data. 

**Figure 8:**
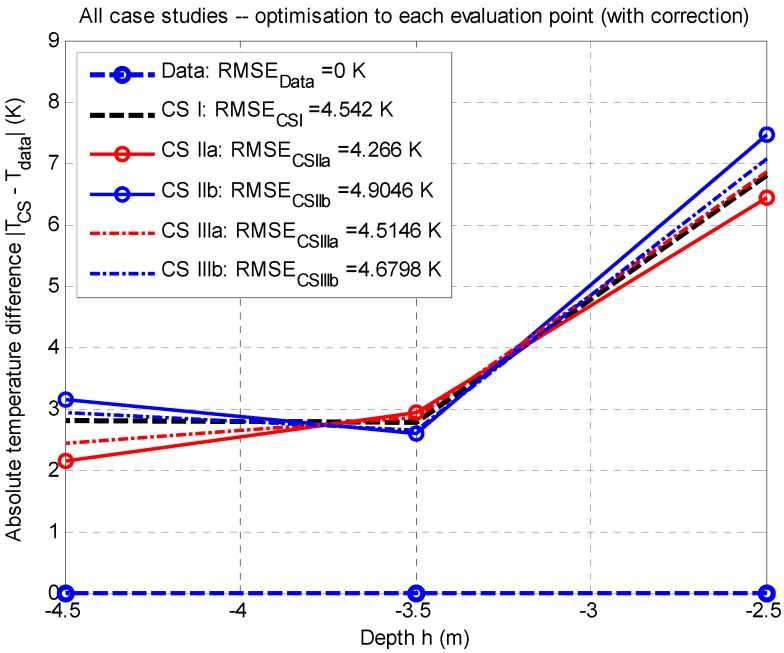
All case studies: Comparison of the results for point optimization (and correction to known values of the thermodynamic temperature at a given evaluation point) to measured data (reference value ${\mathrm{RMSE}}_{\mathrm{Data}}=0\;K$) via the use of the mean-square error RMSE (Kelvins); stationary job, 2D model.

**Figure 9:**
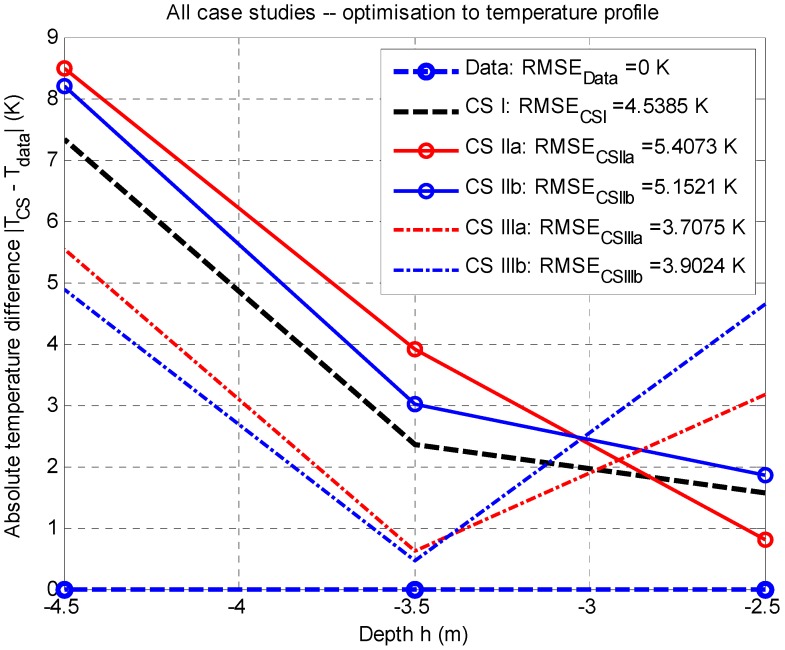
All case studies: Comparison of the results for the thermal profile to measured data (reference value ${\mathrm{RMSE}}_{\mathrm{Data}}=0\;K$) via the use of the mean-square error RMSE (Kelvins); stationary job, 2D model.

For a comparison of the results in all case studies, the mean-square error of the difference model data and measured data, the RMSE (in K, see Figure 8 and Figure 9), was used as an evaluation criterion; it was also possible to determine the area under a curve S (in K∙m). In both cases, the evaluation proved that the lower the value, the better match between the model and data, see Figure 8 and Figure 9.

Based on Figure 8, it is obvious that the lowest value of the mean-square error occurs in case study CS IIa (see Figure 4, Table 6 and Table 7), having the value of ${\mathrm{RMSE}}_{\mathrm{CS}\;\mathrm{IIa}}=4.2660\;K$ (corresponding area under a curve $S_{\mathrm{CS}\;\mathrm{IIa}}=7.2346\;K\cdot m$) at the averaged corrected value $\bar{{\dot{Q}}_{\mathrm{source},\mathrm{corr}.}}=+234.2039\;W$. The local minimum of the objective function, according to Equation (16), is for each evaluation point $f_{\min,B}\left(x\right)=6.8510\;K$, $f_{\min,C}\left(x\right)=4.0580\;K$, and $f_{\min,D}\left(x\right)=0.4124\;K$ (with penalization); in summary, with respect to Equation (17), it is $f_{\min}\left(x\right)=11.3214\;K$ (see also Figure 2).

Case study CS I (see Figure 3, Table 4 and Table 5) shows the mean-square error of ${\mathrm{RMSE}}_{\mathrm{CS}\;I}=4.5420\;K$ (corresponding area under a curve $S_{\mathrm{CS}\;I}=7.5751\;K\cdot m$), at the averaged corrected value $\bar{{\dot{Q}}_{\mathrm{source},\mathrm{corr}.}}=+220.0059\;W$. The local minimum of the objective function, according to Equation (16), is $f_{\min,B}\left(x\right)=4.0199\;K$, $f_{\min,C}\left(x\right)=5.9740\;K$, and $f_{\min,D}\left(x\right)=0.0669\;K$ for each evaluation point; in summary, with respect to Equation (17), it is $f_{\min}\left(x\right)=10.0608\;K$ (see also Figure 2).

Based on Figure 9, it is obvious that the lowest value of the mean-square error occurs in case study CS IIIa (see Table 11), having the value of ${\mathrm{RMSE}}_{\mathrm{CS}\;\mathrm{IIIa}}=3.7075\;K$ (corresponding area under a curve $S_{\mathrm{CS}\;\mathrm{IIIa}}=4.9698\;K\cdot m$) at the estimated value ${\dot{Q}}_{\mathrm{source},\mathrm{est}._{\left(B,C,D\right)}}=+223.7029\;W$. The local minimum of the objective function, according to Equation (17), works out at $f_{\min}\left(x\right)=9.3710\;K$.

Case study CS I (see Table 5) has the mean-square error ${\mathrm{RMSE}}_{\mathrm{CS}\;I}=4.5385\;K$ (corresponding area under a curve $S_{\mathrm{CS}\;I}=6.7995\;K\cdot m$) at the estimated value ${\dot{Q}}_{\mathrm{source},\mathrm{est}._{\left(B,C,D\right)}}=+215.5451\;W$. The local minimum of the objective function, according to Equation (17), works out at $f_{\min}\left(x\right)=11.2800\;K$.

## 4. Discussion

This Section gives a discussion of the importance of the optimization approaches (i.e., point optimization and optimization of the temperature profile; see the results of individual studies in Section 3.1) and the choice of the most appropriate value of the positive heat source based on the type of optimization, the size of the objective function (see Equations (16) and (17)), the size of the mean-square error, possibly also the size of the area under a curve (see Figure 8 and Figure 9), and the balance of heat sources (i.e., positive versus negative values; see Equation (2)).

Generally, the structure of the case studies is based on the nature of the nominal values of the input variables (see case study I in Section 3.1.1), or the method of its determination, either based on the real measured input data (see Case study III in Section 3.1.3) or based on estimates (see Case study II in Section 3.1.2) in cases where no specific data are available. Each study examines its influence on the resulting value.

### 4.1. Discussion for Optimization to The Evaluation Points

The concept of optimization applied for the case studies in this paper can be summed up as follows:

- Effects of the case study I: It is a case study considering nominal values of negative heat sources (see Section 3.1.1). The vector of limits L (Watts) plays an important role in this case study. In the case of its inappropriate setting (for example the localization of the optimized value at the edge of the range), the best estimate of the optimized value (i.e., the value of an objective function) is usually too high; this phenomenon can be observed at values of negative heat sources ${\dot{Q}}_{1}$ and ${\dot{Q}}_{4}$ (Watts) set up at 110% of the reference values (see Section “Case study IIa: The influence of a +10% increase of reference values.”).
- Effects of other case studies: If the values of the negative heat sources ${\dot{Q}}_{2}$ and ${\dot{Q}}_{3}$ (Watts) are set up at 90% of their reference values (values based on measured data; see Section “Case Study IIIb: The Influence of −10% of Reference Values”), their relative errors are lower compared to relative errors related to values of the negative heat sources ${\dot{Q}}_{1}$ and ${\dot{Q}}_{4}$ (Watts), which are set up at 90% of their reference values (estimated values; see Section “Case Study IIb: The Influence of −10% of Reference Values”). The same conclusion can be also stated for values of the heat sources ${\dot{Q}}_{2}$ and ${\dot{Q}}_{3}$ (Watts) set up at 110% of their reference value (see Section “Case Study IIIa: The Influence of +10% of reference values”) and for ${\dot{Q}}_{1}$ and ${\dot{Q}}_{4}$ (Watts), which are set up at 110% of their reference value (see Section “Case study IIa: The influence of a +10% increase of reference values.”).
- The sensitivity of the model: It can be said that the model is more sensitive to the estimated value than the value of the measured data (see case study IIa), namely by a ten percent increase of estimates. A ten percent decrease in the measured data will not cause large variations for ${\dot{Q}}_{\mathrm{source}}$, which is desirable.
- Relative errors: They are related to the reference values of negative heat sources and for individual case studies reveal the percentage decrease of the increase of the relative error compared to the reference. The magnitude of this error is directly proportional to the absolute difference of all positive heat sources: the smaller it is, the better the model responds to deviations of input (typically measurement data). The lowest relative error values to corrected values (i.e., from −1.8935% to +0.6095%; see Table 13) is shown by the case study IIIb (see Section “Case Study IIIb: The Influence of −10% of Reference Values”), where the heat sources ${\dot{Q}}_{2}$ and ${\dot{Q}}_{3}$ are adjusted to 90% of the reference values. In contrast, the highest values of relative error compared to corrected values (i.e., from −2.4563% to +1.5240%; see Table 7) are shown by the case study IIa (see Section “Case study IIa: The influence of a +10% increase of reference values.”), where ${\dot{Q}}_{1}$ and ${\dot{Q}}_{4}$ are adjusted to 110% of their reference values.
- Effects of correction: Regarding this optimization approach, this correction is manifested by small (and comparable) differences between the mean-square error (or area under a curve, see Figure 8) for individual case studies, which is not always advantageous. The behavior at nominal values may not be the best from a given set of available solutions, and it is therefore necessary to test the input data (see Section 3.1).
- Optimal solution: Considering the value of the mean-square error, this is related to case study IIa (see Section “Case study IIa: The influence of a +10% increase of reference values.”), i.e., 110% of the reference value of the negative heat sources ${\dot{Q}}_{1,\mathrm{CS}\;\mathrm{IIa}}$ and ${\dot{Q}}_{4,\mathrm{CS}\;\mathrm{IIa}}$, and the nominal value of ${\dot{Q}}_{2,\mathrm{CS}\;\mathrm{IIa}}$ and ${\dot{Q}}_{3,\mathrm{CS}\;\mathrm{IIa}}$ for evaluation points B, C, and D (see Table 6 and Table 7). Without correction, this case study was not the optimal solution (see above) due to the higher sensitivity of the model to estimates of temperature differences in Equations (11) and (14) when considering the validity of Equation (10).

The reason for carrying out the correction is given by the fact that both point optimization and temperature-profile optimization without correction do not guarantee the determination of the positive heat source at the desired temperature at a given evaluation point (see $T_{\mathrm{data}}$ in Figure 2). From the application point of view, the correction can be basically performed in two ways:

- Approach I: Correction is based on approximation of the function $\dot{Q}=f\left(T\right)$; see also Section 2.2.5 and Figure 3, Figure 4, Figure 5, Figure 6, Figure 7. Within this paper, the linearization part is defined by the vector $L=\left\{x_{0},{\;x}_{\mathrm{lower}},{\;x}_{\mathrm{upper}}\right\}$. The disadvantage of such linearization is that a plot may be substantially nonlinear. The number of steps in this approach is always one.
- Approach II: This approach is a generalization of Approach I. The above-mentioned disadvantage is eliminated by carrying out a local approximation in the range $\left[x_{\mathrm{lower}}^{*},{\;x}_{\mathrm{upper}}^{*}\right]$, where the supposed optimized value of the positive heat source is located. The simplest local approximation can also be the linearization in the operating point. The disadvantage of this approach is a necessity of carrying out two calculations: first, a determination of the operating point (i.e., the optimized value of the positive heat source ${\dot{Q}}_{\mathrm{source},\mathrm{est}.}$) from the vector $L=\left\{x_{0},{\;x}_{\mathrm{lower}},{\;x}_{\mathrm{upper}}\right\}$, and second a specification of ${\dot{Q}}_{\mathrm{source},\mathrm{est}.}^{*}$ from the vector $L^{*}=\left\{x_{0}^{*}={\dot{Q}}_{\mathrm{source},\mathrm{est}.},{\;x}_{\mathrm{lower}}^{*},{\;x}_{\mathrm{upper}}^{*}\right\}$. The number of steps in this approach is at least two.

### 4.2. Discussion for Optimization to The Temperature Profile

The concept of optimization applied for the case studies in this paper can be summed up as follows:

- Effect of other studies: The structure of the individual case studies is the same as in the case of point optimization.
- The sensitivity of the model: It can be said that it is the same as for point optimization; however, differences (between data and estimates) are more significant (see Figure 9).
- Relative errors: Due to the absence of a correction algorithm, the calculation of corrected values of the positive heat source and the individual relative errors is not necessary.
- Effect of correction: None, since the correction in this case is not performed.
- Optimal solution: Considering the value of the mean-square error, this is related to case study IIIa (see section “Case Study IIIa: The Influence of +10% of reference values”), i.e., 110% of reference values of the negative heat sources ${\dot{Q}}_{2,\mathrm{CS}\;\mathrm{IIIa}}$ and ${\dot{Q}}_{3,\mathrm{CS}\;\mathrm{IIIa}}$ and nominal values of ${\dot{Q}}_{1,\mathrm{CS}\;\mathrm{IIIa}}$ and ${\dot{Q}}_{4,\mathrm{CS}\;\mathrm{IIIa}}$ for the evaluation points B, C, and D (see Table 10 and Table 11). This study represents the best-optimized solution because it shows the lowest mean-square error (i.e., ${\mathrm{RMSE}}_{\mathrm{CS}\;\mathrm{IIIa}}=3.7075\;K$), the lowest area under a curve (i.e., $S_{\mathrm{CS}\;\mathrm{IIIa}}=4.9698\;K\cdot m$), the lowest value of local minima [i.e., $f_{\min}\left(x\right)=9.3710\;K$; with no penalization, and according to Equation (17)], and also the best balance of heat sources [i.e., ${\dot{Q}}_{\mathrm{source},\mathrm{est}._{\left(B,C,D\right)}}+\sum_{i=1}^{4}{\dot{Q}}_{m,\mathrm{CS}\;\mathrm{IIIa}}=\left[+223.7029+\left(-209.1363\right)\right]\;W=14.5666\;W$; considering Equation (2)].

### 4.3. Discussion for Other Input Parameters

Considering the characteristics of the geological environment at the Hedwig dump (see Section “Geological Viewpoint”), it is possible to carry out a parametric sweep (see Table 3) applied for the specific heat capacity within the positive heat source domain, $c_{p,s}$ (J∙kg^−1^∙K^−1^; see Table 2), and the coefficient of thermal conductivity of the geological environment, $\lambda_{s}$ (W∙m^−1^∙K^−1^; see Table 2), and thus to investigate the correctness of the estimate of the material composition of the geological environment based on available documentation (see [10], Section 2.2.1) in the case where no detailed geological report is available for the surroundings of the heat collector.

## 5. Conclusions

The Hedwig mining dump is spread around an area measuring 32.0 × 10^4^ m^2^ (see Table 1), and in terms of its composition it is not principally homogeneous in different parts because the centers of fires of tailings are demarcated (see the temperature distribution in [1]). Related to this, [10] also contains a mixture of rocks and their percentage composition for possible modeling of thermal processes; parameters of surrounding waste rock were determined on this basis [see Table 2 and Equation (1)] to be used in the COMSOL numerical model of the heat collector. This simplification was used in all the case studies (see Section 3.1); neither the effect of variable percentages nor different compositions of the rocks were considered in the evaluation. The structure and geometry of the heat collector itself (i.e., the “Pershing” system) are given in [23] and Figure 1; within data preprocessing, the paper also describes mutual interactions between this structure (where all negative heat sources may be included) and the respective measured input data from the chambers and data from the evaluation points [see Figure 2 and Equations (10) to (14)], which is beneficial. Let us add that in Figure 2, the particular stages of processing the data (i.e., “preprocessing”, “processing”, and “post-processing”) are clearly marked.

When designing and implementing the optimization job (see also Figure 2: the “processing” part), the authors came up with the basic presumption of Equation (2), i.e., the balance of the negative heat sources and positive heat source in steady-state. From an implementation point of view, the authors used a 2D COMSOL model, Matlab script (containing the optimization algorithm itself: the fmincon function and form of the objective function). The form of the objective function given by Equation (16) represents a static optimization evaluation criterion for the evaluation point (i.e., point optimization), while the form of Equation (17) is a static optimization criterion based on the temperature profile. This categorization has proved to be significant because it helped to introduce other alternatives in each case study (i.e., the one related to the temperature profile) and revealed the positives and negatives of both of these optimization approaches (see Section 4.1 and Section 4.2) in the evaluation and validation.

The basis of the evaluation results comprises a total of five case studies (see Section 3.1) relying on the susceptibility testing of the COMSOL model via input data from the evaluation points given by coordinates in the Cartesian coordinate system (see also Figure 2). The authors thus tested how that model will behave in terms of measured data from individual chambers [see Equations (12) and (13)] and in terms of estimated temperature differences [see Equations (11) and (14)]. It turned out that the model was more sensitive to the estimates of the temperature differences (i.e., 90% and 110% of the nominal values in Table 4) where it achieved worse results (see case studies IIa and IIb) when compared with measured data from the chambers (see case studies IIIa and IIIb). It was shown that this observation applies irrespective of the chosen optimization approach.

As part of postprocessing in point optimization (see Figure 2), the authors used an optimized correction value of the positive heat source for the appropriate temperature from the measured data in the individual evaluation points. In Section 4.1, the authors discussed two possible approaches regarding the implementation of this correction; the paper dealt with Approach I. The validation by the use of the mean-square error (see Figure 8 and Figure 9) showed that the application of a correction value ensured a favorable heat source at the desired temperature, but that for the point optimization it almost eliminated differences between case studies (see Figure 8), and this fact made it harder to find the optimal solution when compared to temperature profile-based optimization (see Figure 9). The optimal solution was found in case study IIIa (see Section “Case Study IIIb: The Influence of −10% of Reference Values”) considering the thermal profile-based approach, and it satisfied the criteria of the lowest value of mean-square error, the lowest value of the local minimum of the objective function, and the best balance of heat sources.

This paper emphasized the description of the proposed solution and algorithm proposed as giving a guess of one of the crucial parameters related to the mining dumps, which is the value of the heat source represented by the mining dump.

These innovative outcomes can be summarized as follows:

- Connection of FEM modeling tool and measured data waveforms to evaluate optimization job
- Possibility to compute an estimated value of the total heat capacity of slag heaps
- The proposed experiment with temperature probes and low requirements on hardware and software setup

The authors have achieved their set goals. Regarding future work, there are the following plans:

- Completion of the COMSOL model in terms of the precise definition of the boundary conditions at the edges of the individual domains (see Figure 1 and Table 3); the only boundary condition is represented by Equation (8) without further association.
- Carrying out a study dealing with the transient problem [see also Equation (15)], based on a methodological procedure presented in this article and valid solutions for stationary tasks.

## Figures and Tables

**Table 1:** The Hedwig mining dump–basic technical parameters (see also [10]).

Parameter	Value	Unit
dump’s soil volume	4.2 × 10^6^	m^3^
dump’s area	32.0 × 10^4^	m^2^
the average thickness of the loose body	15	m
maximal thickness of the loose body	40 (locally)	m
Dump’s operation period	1900–1998	------
the technology of transporting an excavated material	using mine trucks	------
the technology of storing excavated material	successive dumping into a valley system	------

**Table 2:** Mathematical model—values of the chosen physical parameters in the target object Heat Source 5 based on the percentage content of particular rocks.

Parameter	Value							Unit
	Sandstone		Siltstone		Claystone		Result	
	$p_{1}$	------	$p_{2}$	------	$p_{3}$	------		
$\lambda_{s}$	0.18	2.000	0.50	2.300	0.32	2.200	2.214	W∙m^−1^∙K^−1^
$\rho_{s}$		2500		2600		2600	2582	kg∙m^−3^
$\rho_{s}\cdot c_{p,s}$		2.000 × 10^6^		2.300 × 10^6^		2.300 × 10^6^	2.246 × 10^6^	J∙m^−3^∙K^−1^
$c_{p,s}$		800.000		884.615		884.615	869.868	J∙kg^−1^∙K^−1^
$a_{s}$		1.000 × 10^−6^		1.000 × 10^−6^		0.957 × 10^−6^	0.986 × 10^−6^	m^2^∙s^−1^

**Table 3:** Mathematical model—globally defined parameters of the model in the form of heat sources and their implementation in COMSOL (i.e., the heat sources, domains, and target objects).

Heat Sources		Domains			Target Object
Parameter	Unit	ID	Volume	Unit	
${\dot{Q}}_{1}$	W	3	50.9181 × 10^−3^	m^3^	Heat Source 1
${\dot{Q}}_{2}$	W	4 and 7	50.7218 × 10^−3^	m^3^	Heat Source 2
${\dot{Q}}_{3}$	W	5 and 8	50.7218 × 10^−3^	m^3^	Heat Source 3
${\dot{Q}}_{4}$	W	6 and 9	50.7218 × 10^−3^	m^3^	Heat Source 4
${\dot{Q}}_{5}\equiv {\dot{Q}}_{\mathrm{source}}$	W	1 and 11	86.2254	m^3^	Heat Source 5

**Table 4:** The case study I: Reference values of the negative heat sources ${\dot{Q}}_{1,\mathrm{CS}\;I}$ to ${\dot{Q}}_{4,\mathrm{CS}\;I}$, and estimations of the positive heat source ${\dot{Q}}_{\mathrm{source},\mathrm{est}.}$ for evaluation points B, C, and D.

Evaluation Points	Heat Sources				
	${\dot{Q}}_{1,\mathrm{CS}\;I}$	${\dot{Q}}_{2,\mathrm{CS}\;I}$	${\dot{Q}}_{3,\mathrm{CS}\;I}$	${\dot{Q}}_{4,\mathrm{CS}\;I}$	${\dot{Q}}_{\mathrm{source},\mathrm{est}.}$
(m)	(W)	(W)	(W)	(W)	(W)
B: [0.5; −2.5]	−5.2377	−12.0464	−52.3756	−133.0341	+213.6783
C: [0.5; −3.5]					+213.6802
D: [0.5; −4.5]					+224.0250
-----	$\overset{4}{\underset{m=1}{\sum }}{\dot{Q}}_{m,\mathrm{CS}\;I}=-202.6938\;W$				-----

Number of samples: N=684. Vector of constrained limits: $L=\left\{x_{0},{\;x}_{\mathrm{lower}},{\;x}_{\mathrm{upper}}\right\}=\left\{200,\;100,\;250\right\}\;W$.

**Table 5:** The case study I: Estimation of the positive heat source ${\dot{Q}}_{\mathrm{source},\mathrm{est}.}$, and consequent corrections ${\dot{Q}}_{\mathrm{source},\mathrm{corr}.}$ to thermodynamic temperatures $\bar{T_{B}}$, $\bar{T_{C},}$ and $\bar{T_{D}}$ and for evaluation points B, C, and D (for points and a thermal profile).

Evaluation Points	Optimization		Correction		
	${\dot{Q}}_{\mathrm{source},\mathrm{est}.}$	$\delta_{\mathrm{CS}\;I,\mathrm{est}.}$	${\dot{Q}}_{\mathrm{source},\mathrm{corr}.}$	$\delta_{\mathrm{CS}\;I,\mathrm{corr}.}$	T
(m)	(W)	(%)	(W)	(%)	(K)
B: [0.5; −2.5]	+213.6783	−1.5392	+217.0187	0	293.6104
C: [0.5; −3.5]	+213.6802	−2.3879	+218.9076	0	306.3205
D: [0.5; −4.5]	+224.0250	−0.0296	+224.0914	0	314.9920
B, C, D	+215.5451	-----	-----	-----	Temperature profile

All thermodynamic temperatures for the thermal profile: $\bar{T_{B}}=293.6104\;K$, $\bar{T_{C}}=306.3205\;K$, and $\bar{T_{D}}=314.9920\;K$.

**Table 6:** Case study IIa: 110% of the reference value of the negative heat sources ${\dot{Q}}_{1,\mathrm{CS}\;\mathrm{IIa}}$ and ${\dot{Q}}_{4,\mathrm{CS}\;\mathrm{IIa}}$, and the nominal values of the heat sources ${\dot{Q}}_{2,\mathrm{CS}\;\mathrm{IIa}}$ and ${\dot{Q}}_{3,\mathrm{CS}\;\mathrm{IIa}}$ for evaluation points B, C, and D.

Evaluation Points	Heat Sources				
	${\dot{Q}}_{1,\mathrm{CS}\;\mathrm{IIa}}$	${\dot{Q}}_{2,\mathrm{CS}\;\mathrm{IIa}}$	${\dot{Q}}_{3,\mathrm{CS}\;\mathrm{IIa}}$	${\dot{Q}}_{4,\mathrm{CS}\;\mathrm{IIa}}$	${\dot{Q}}_{\mathrm{source},\mathrm{est}.}$
(m)	(W)	(W)	(W)	(W)	(W)
B: [0.5; −2.5]	−5.7615	−12.0464	−52.3756	−146.3375	+226.0771
C: [0.5; −3.5]					+236.5147
D: [0.5; −4.5]					+238.2865
-----	$\overset{4}{\underset{m=1}{\sum }}{\dot{Q}}_{m,\mathrm{CS}\;\mathrm{IIa}}=-216.5210\;W$				-----

The number of samples: N=684. Vector of constrained limits: $L=\left\{x_{0},{\;x}_{\mathrm{lower}},{\;x}_{\mathrm{upper}}\right\}=\left\{200,\;100,\;250\right\}\;W$.

**Table 7:** Case study IIa: Estimations of the positive heat source ${\dot{Q}}_{\mathrm{source},\mathrm{est}.}$ and consequent corrections ${\dot{Q}}_{\mathrm{source},\mathrm{corr}.}$ to thermodynamic temperatures $\bar{T_{B}}$, $\bar{T_{C}}$, and $\bar{T_{D}}$ for evaluation points B, C, and D (for points and a thermal profile).

Evaluation Points	Optimization		Correction		
	${\dot{Q}}_{\mathrm{source},\mathrm{est}.}$	$\delta_{\mathrm{CS}\;\mathrm{IIa},\mathrm{est}.}$	${\dot{Q}}_{\mathrm{source},\mathrm{corr}.}$	$\delta_{\mathrm{CS}\;\mathrm{IIa},\mathrm{corr}.}$	T
(m)	(W)	(%)	(W)	(%)	(K)
B: [0.5; −2.5]	+226.0771	−2.4563	+231.7701	+6.7973	293.6104
C: [0.5; −3.5]	+236.5147	+1.5240	+232.9643	+6.4213	306.3205
D: [0.5; −4.5]	+238.2865	+0.1721	+237.8772	+6.1519	314.9920
B, C, D	+228.2198	-----	-----	-----	Temperature profile

All thermodynamic temperatures for the temperature profile: $\bar{T_{B}}=293.6104\;K$, $\bar{T_{C}}=306.3205\;K$, and $\bar{T_{D}}=314.9920\;K$.

**Table 8:** Case study IIb: 90% of reference values of the negative heat sources ${\dot{Q}}_{1,\mathrm{CS}\;\mathrm{IIb}}$ and ${\dot{Q}}_{4,\mathrm{CS}\;\mathrm{IIb}}$ and the nominal values of the heat sources ${\dot{Q}}_{2,\mathrm{CS}\;\mathrm{IIb}}$ a ${\dot{Q}}_{3,\mathrm{CS}\;\mathrm{IIb}}$ for evaluation points B, C, and D.

Evaluation Points	Heat Sources				
	${\dot{Q}}_{1,\mathrm{CS}\;\mathrm{IIb}}$	${\dot{Q}}_{2,\mathrm{CS}\;\mathrm{IIb}}$	${\dot{Q}}_{3,\mathrm{CS}\;\mathrm{IIb}}$	${\dot{Q}}_{4,\mathrm{CS}\;\mathrm{IIb}}$	${\dot{Q}}_{\mathrm{source},\mathrm{est}.}$
(m)	(W)	(W)	(W)	(W)	(W)
B: [0.5; −2.5]	−4.7139	−12.0464	−52.3756	−119.7307	+201.1988
C: [0.5; −3.5]					+201.1384
D: [0.5; −4.5]					+211.3246
-----	$\overset{4}{\underset{m=1}{\sum }}{\dot{Q}}_{m,\mathrm{CS}\;\mathrm{IIb}}=-188.8666\;W$				-----

The number of samples: N=684. Vector of constrained limits: $L=\left\{x_{0},{\;x}_{\mathrm{lower}},{\;x}_{\mathrm{upper}}\right\}=\left\{200,\;100,\;250\right\}\;W$.

**Table 9:** Case study IIb: Estimation of the positive heat source ${\dot{Q}}_{\mathrm{source},\mathrm{est}.}$ and consequent corrections ${\dot{Q}}_{\mathrm{source},\mathrm{corr}.}$ to thermodynamic temperatures $\bar{T_{B}}$, $\bar{T_{C}}$, and $\bar{T_{D}}$ for evaluation points B, C, and D (for points and a thermal profile).

Evaluation Points	Optimization		Correction		
	${\dot{Q}}_{\mathrm{source},\mathrm{est}.}$	$\delta_{\mathrm{CS}\;\mathrm{IIb},\mathrm{est}.}$	${\dot{Q}}_{\mathrm{source},\mathrm{corr}.}$	$\delta_{\mathrm{CS}\;\mathrm{IIb},\mathrm{corr}.}$	T
(m)	(W)	(%)	(W)	(%)	(K)
B: [0.5; −2.5]	+201.1988	−0.5283	+202.2673	−6.7973	293.6104
C: [0.5; −3.5]	+201.1384	−1.8122	+204.8508	−6.4213	306.3205
D: [0.5; −4.5]	+211.3246	+0.4846	+210.3055	−6.1519	314.9920
B, C, D	+200.9861	-----	-----	-----	Temperature profile

All thermodynamic temperatures for the temperature profile: $\bar{T_{B}}=293.6104\;K$, $\bar{T_{C}}=306.3205\;K$, and $\bar{T_{D}}=314.9920\;K$.

**Table 10:** Case study IIIa: 110% of the reference values of the negative heat sources ${\dot{Q}}_{2,\mathrm{CS}\;\mathrm{IIIa}}$ and ${\dot{Q}}_{3,\mathrm{CS}\;\mathrm{IIIa}}$ and the nominal values of the heat sources ${\dot{Q}}_{1,\mathrm{CS}\;\mathrm{IIIa}}$ and ${\dot{Q}}_{4,\mathrm{CS}\;\mathrm{IIIa}}$ for evaluation points B, C, and D.

Evaluation Points	Heat Sources				
	${\dot{Q}}_{1,\mathrm{CS}\;\mathrm{IIIa}}$	${\dot{Q}}_{2,\mathrm{CS}\;\mathrm{IIIa}}$	${\dot{Q}}_{3,\mathrm{CS}\;\mathrm{IIIa}}$	${\dot{Q}}_{4,\mathrm{CS}\;\mathrm{IIIa}}$	${\dot{Q}}_{\mathrm{source},\mathrm{est}.}$
(m)	(W)	(W)	(W)	(W)	(W)
B: [0.5; −2.5]	−5.2377	−13.2510	−57.6135	−133.0341	+223.3922
C: [0.5; −3.5]					+225.2415
D: [0.5; −4.5]					+226.6173
-----	$\overset{4}{\underset{m=1}{\sum }}{\dot{Q}}_{m,\mathrm{CS}\;\mathrm{IIIa}}=-209.1363\;W$				-----

The number of samples: N=684. Vector of constrained limits: $L=\left\{x_{0},{\;x}_{\mathrm{lower}},{\;x}_{\mathrm{upper}}\right\}=\left\{200,\;100,\;250\right\}\;W$.

**Table 11:** Case study IIIa: Estimation of the positive heat source ${\dot{Q}}_{\mathrm{source},\mathrm{est}.}$ and consequent corrections ${\dot{Q}}_{\mathrm{source},\mathrm{corr}.}$ to thermodynamic temperatures $\bar{T_{B}}$, $\bar{T_{C}}$, and $\bar{T_{D}}$ for evaluation points B, C, and D (for points and a thermal profile).

Evaluation Points	Optimization		Correction		
	${\dot{Q}}_{\mathrm{source},\mathrm{est}.}$	$\delta_{\mathrm{CS}\;\mathrm{IIIa},\mathrm{est}.}$	${\dot{Q}}_{\mathrm{source},\mathrm{corr}.}$	$\delta_{\mathrm{CS}\;\mathrm{IIIa},\mathrm{corr}.}$	T
(m)	(W)	(%)	(W)	(%)	(K)
B: [0.5; −2.5]	+223.3922	−0.2407	+223.9313	+3.1852	293.6104
C: [0.5; −3.5]	+225.2415	−0.1543	+225.5895	+3.0524	306.3205
D: [0.5; −4.5]	+226.6173	−1.7047	+230.5475	+2.8810	314.9920
B, C, D	+223.7029	-----	-----	-----	Temperature profile

All thermodynamic temperatures for the temperature profile: $\bar{T_{B}}=293.6104\;K$, $\bar{T_{C}}=306.3205\;K$, and $\bar{T_{D}}=314.9920\;K$.

**Table 12:** Case study IIIb: 90% of the reference values of the negative heat sources ${\dot{Q}}_{2,\mathrm{CS}\;\mathrm{IIIb}}$ and ${\dot{Q}}_{3,\mathrm{CS}\;\mathrm{IIIb}}$ and the nominal values of the heat sources ${\dot{Q}}_{1,\mathrm{CS}\;\mathrm{IIIb}}$ and ${\dot{Q}}_{4,\mathrm{CS}\;\mathrm{IIIb}}$ for evaluation points B, C, and D.

Evaluation Points	Heat Sources				
	${\dot{Q}}_{1,\mathrm{CS}\;\mathrm{IIIb}}$	${\dot{Q}}_{2,\mathrm{CS}\;\mathrm{IIIb}}$	${\dot{Q}}_{3,\mathrm{CS}\;\mathrm{IIIb}}$	${\dot{Q}}_{4,\mathrm{CS}\;\mathrm{IIIb}}$	${\dot{Q}}_{\mathrm{source},\mathrm{est}.}$
(m)	(W)	(W)	(W)	(W)	(W)
B: [0.5; −2.5]	−5.2377	−10.8418	−47.1380	−133.0341	+211.3872
C: [0.5; −3.5]					+212.6770
D: [0.5; −4.5]					+213.5146
-----	$\overset{4}{\underset{m=1}{\sum }}{\dot{Q}}_{m,\mathrm{CS}\;\mathrm{IIIb}}=-196.2516\;W$				-----

The number of samples: N=684. Vector of constrained limits: $L=\left\{x_{0},{\;x}_{\mathrm{lower}},{\;x}_{\mathrm{upper}}\right\}=\left\{200,\;100,\;250\right\}\;W$.

**Table 13:** Case study IIIb: Estimation of the positive heat source ${\dot{Q}}_{\mathrm{source},\mathrm{est}.}$ and consequent corrections ${\dot{Q}}_{\mathrm{source},\mathrm{corr}.}$ to thermodynamic temperatures $\bar{T_{B}}$, $\bar{T_{C}}$, and $\bar{T_{D}}$ for evaluation points B, C, and D (for points and a thermal profile).

Evaluation Points	Optimization		Correction		
	${\dot{Q}}_{\mathrm{source},\mathrm{est}.}$	$\delta_{\mathrm{CS}\;\mathrm{IIIb},\mathrm{est}.}$	${\dot{Q}}_{\mathrm{source},\mathrm{corr}.}$	$\delta_{\mathrm{CS}\;\mathrm{IIIb},\mathrm{corr}.}$	T
(m)	(W)	(%)	(W)	(%)	(K)
B: [0.5; −2.5]	+211.3872	+0.6095	+210.1065	−3.1851	293.6104
C: [0.5; −3.5]	+212.6770	+0.2125	+212.2260	−3.0522	306.3205
D: [0.5; −4.5]	+213.5146	−1.8935	+217.6356	−2.8809	314.9920
B, C, D	+211.4523	-----	-----	-----	Temperature profile

All thermodynamic temperatures for the temperature profile: $\bar{T_{B}}=293.6104\;K$, $\bar{T_{C}}=306.3205\;K$, and $\bar{T_{D}}=314.9920\;K$.

## References

[1] Hajovsky R., Filipova B., Pies M., Ozana S. Using Matlab for thermal processes modeling and prediction at mining dumps. Proceedings of the International Conference on Control, Automation and Systems 2012.

[2] Klika Z., Kraussová J. (1993). Properties of altered coals associated with carboniferous red beds in the upper silesian coal basin and their tentative classification. Int. J. Coal Geol..

[3] Klika Z. (1998). Geochemistry of Coal from Region of the Red Beds Bodies of the Upper Silesian Coal Basin.

[4] Pryor R.W. (2011). Multiphysics Modeling using COMSOL: A First Principles Approach.

[5] Price M., Walsh K. (2005). H Rocks & Minerals (Pocket Nature).

[6] Výpis z databáze patentů a Užitných Vzorů. Isdv.upv.cz/portal/pls/portlets.pts.det?xprim=2079157&lan=cs&s_majs=&s_puvo=štěpánožana&s_naze=&s=anot.

[7] Wessling S., Kuenzer C., Kessels W., Wuttke M.W. (2008). Numerical modeling for analyzing thermal surface anomalies induced by underground coal fires. Int. J. Coal Geol..

[8] Rohsenow W.M., Hartnett J.P., Cho Y.I. (1998). Handbook of Heat Transfer.

[9] Hower J.C., O’Keefe J.M.K., Henke K.P., Wagner N.J., Copley G., Blake D.R., Garrison T., Oliveira M.L.S., Kautzmann R.M., Silva L.F.O. (2013). Gaseous emissions and sublimates from the Truman Shepherd coal fire, Floyd County, Kentucky: A re-investigation following attempted mitigation of the fire. Int. J. Coal Geol..

[10] Kresta F. (2013). Enhancement of Quality of Environment with Respect to Occurrence of Endogenous Fires in Mine Dumps and Industrial Waste Dumps, Including its Modeling and Spread Prediction.

[11] Nie X., Wei X., Li X., Lu C. (2018). Heat treatment and ventilation optimization in a deep mine. Adv. Civ. Eng..

[12] Lai J., Qiu J., Fan H., Chen J., Xie Y. (2016). Freeze-proof method and test verification of a cold region tunnel employing electric heat tracing. Tunn. Undergr. Space Technol..

[13] Ozana S., Pies M., Hajovsky R. (2014). Using MATLAB and COMSOL multiphysics for optimization of the model of underground thermal processes at old mining dumps. Appl. Mech. Mater..

[14] Hajovsky R., Pies M. (2016). Experience in collecting heat at the Hedvika and Krimich thermally active mining dumps. IFAC Pap..

[15] Hajovsky R., Hajovsky J., Pies M. (2013). Thermall processes at old mining dumps and their measurement and utilization. Lect. Notes Eng. Comput. Sci..

[16] Huang J., Bruining J., Wolf K.H.A.A. (2001). Modeling of gas flow and temperature fields in underground coal fires. Fire Saf. J..

[17] Klika Z., Kozubek T., Martinec P., Kliková C., Dostál Z. (2004). Mathematical modeling of bituminous coal seams burning contemporaneously with the formation of a variegated beds body. Int. J. Coal Geol..

[18] Zhang J., Kuenzer C. (2007). Thermal surface characteristics of coal fires 1: Results of in-situ measurements. J. Appl. Geophys..

[19] Polyanin A.D. (2002). Handbook of Linear Partial Equations for Engineers and Scientists.

[20] Pies M., Ozana S., Hajovsky R., Vojcinak P. (2013). Measurement and simulation of underground heat collecting processes with COMSOL multiphysics. Lect. Notes Eng. Comput. Sci..

[21] Hajovsky R., Pies M., Ozana S., Hajovsky J. Heat energy collection from thermally active mining dump Hedvika. Proceedings of the IEEE International Conference on Automation Science and Engineering.

[22] Klika Z. (2011). Burning Coal Dumps II: Calculation of Heat Balances at the Hedwig Mining Dump.

[23] Ribeiro J., da Silva F.E., Floresa D. (2010). Burning of coal waste piles from duoro coalfield (Portugal): Petrological, geochemical and mineralogical characterization. Int. J. Coal Geol..

[24] COMSOL (2009–2011). LiveLink™ for MATLAB®: User’s Guide.

[25] Houlding S.L. (1994). 3D Geoscience Modeling: Computer Techniques for Geological Characterization.

[26] Demek J., Mackovčin P., Balatka B. (2006). Geographical Lexicon of the Czech Republic: Mountains and Lowlands.

[27] Brázdil R., Kirchner K. (2007). Selected Natural Extremes and their Impacts in Moravia and Silesia.

[28] Geoportál. http://geoportal.gov.cz/web/guest/map.

[29] Bujok P., Grycz D., Klempa M., Kunz A., Porzer M., Pytlík A., Rozehnal Z., Vojčinák P. (2014). Assessment of the influence of shortening the duration of TRT (Thermal Response Test) on the precision of measured values. Energy.

[30] Incropera F.P., DeWitt D.P. (2002). Introduction to Heat Transfer.

[31] Querol X., Izquierdo M., Monfort E., Alvarez E., Font O., Moreno T., Alastuey A., Zhuang X., Lu W., Wang Y. (2008). Environmental characterization of burnt coal gangue banks at Yangquan, Shanxi Province, China. Int. J. Coal Geol..

